# Cholesterol modulates acetylcholine receptor diffusion by tuning confinement sojourns and nanocluster stability

**DOI:** 10.1038/s41598-018-30384-y

**Published:** 2018-08-10

**Authors:** Alejo Mosqueira, Pablo A. Camino, Francisco J. Barrantes

**Affiliations:** Laboratory of Molecular Neurobiology, Biomedical Research Institute (BIOMED), UCA–CONICET, Av. Alicia Moreau de Justo 1600, C1107AFF Buenos Aires, Argentina

## Abstract

Translational motion of neurotransmitter receptors is key for determining receptor number at the synapse and hence, synaptic efficacy. We combine live-cell STORM superresolution microscopy of nicotinic acetylcholine receptor (nAChR) with single-particle tracking, mean-squared displacement (MSD), turning angle, ergodicity, and clustering analyses to characterize the lateral motion of individual molecules and their collective behaviour. nAChR diffusion is highly heterogeneous: subdiffusive, Brownian and, less frequently, superdiffusive. At the single-track level, free walks are transiently interrupted by ms-long confinement sojourns occurring in nanodomains of ~36 nm radius. Cholesterol modulates the time and the area spent in confinement. Turning angle analysis reveals anticorrelated steps with time-lag dependence, in good agreement with the permeable fence model. At the ensemble level, nanocluster assembly occurs in second-long bursts separated by periods of cluster disassembly. Thus, millisecond-long confinement sojourns and second-long reversible nanoclustering with similar cholesterol sensitivities affect all trajectories; the proportion of the two regimes determines the resulting macroscopic motional mode and breadth of heterogeneity in the ensemble population.

## Introduction

Transmission of chemical signals in the synapse is regulated by the crosstalk between neurotransmitter receptors and scaffolding proteins, lipids, and the cytoskeleton. To understand synaptic physiology, it is necessary to define the supramolecular organization, local dynamics and trafficking of the intervening actors. The available number of neurotransmitter receptors and the time they spend in the post-synaptic region and, more precisely, at the actives sites facing the neurotransmitter releasing areas, directly affects synaptic activity. Comprehension of the various phenomena that determine this spatio-temporal homeostatic balance is key to understanding the function of the synapse in health and disease. One such phenomenon is the motion of receptors in the plasma membrane. How receptors diffuse into and out of the synaptic region, and which mechanisms affect lateral diffusion and maintenance of receptors in the post-synaptic region are fundamental to an understanding of synaptic function.

The superfamily of pentameric ligand-gated ion channels (pLGIC) is a collection of integral membrane proteins expressed in the central and peripheral nervous system, where they perform important functions in cell-surface signalling by transducing the chemical signal contained in the neurotransmitter into rapid ion fluxes at the post-synaptic membrane. One of the best characterized neurotransmitter receptors is the nicotinic acetylcholine receptor (nAChR) protein, the paradigm member of the pLGIC, a hot focus of research for possible intervention in neurological and neuropsychiatric diseases. The membrane lipid environment in which receptors are embedded affects the functional properties and distribution of the protein macromolecules. Cholesterol is an abundant component in the post-synaptic membrane, and there is a plethora of evidence on the variety of modulatory roles exerted by this lipid on the nAChR^1^. Interestingly, the members of the pLGIC superfamily are descendants of an ancestral “proto-channel” which appeared early in phylogenetic evolution, before the prokaryote-eukaryote dichotomy, and although almost all prokaryotes lack cholesterol, the pLGIC exhibit the same sterol-recognition motifs as their eukaryotic counterparts^2,3^. This remarkable degree of conservation at the molecular scale points to the importance of cholesterol in pLGIC function. In the specific case of the nAChR, we know that cholesterol exhibits preference over other lipid species for the superficial region surrounding the surface of the receptor protein; about 15 cholesterol molecules appear to be located at this region^4^. These cholesterol molecules are in constant exchange with the bulk bilayer cholesterol^5^, which is a key regulator of physical membrane properties at large. At the subcellular scale, there is also evidence that nAChRs interact with cholesterol-rich lipid domains (“rafts”) *in vitro* and *in vivo*^6–8^. Acute cholesterol depletion reduces the number of receptor domains by accelerating the rate of endocytosis and shifting the internalization to a different endocytic pathway involving the small GTPase Arf6^9^.

Stimulated emission depletion (STED) nanoscopy, i.e. a targeted superresolution microscopy method, has been used to study the static supramolecular organization of the muscle-type nAChR at the cell surface of fixed mammalian cells^10^. In the present work, we employ single-molecule localization microscopy (SMLM) in the form of stochastic optical reconstruction microscopy (STORM) to image live CHO-K1/A5 cells labelled with a monovalent ligand, fluorescent α-bungarotoxin (BTX). We followed the translational dynamics of the nAChR at the cell surface using single-particle tracking (SPT) methods. The experimental data was subjected to an intertwined combination of analytical and statistical techniques, disclosing a wide distribution of diffusivities, with a predominant mixture of subdiffusive and Brownian regimes. The physical models tested to reveal the source of the anomalous diffusion suggest that coexisting macromolecular self-crowding of the nAChR and obstacles in the form of percolating barriers operate in the time scale of tens of milliseconds to seconds, hampering free nAChR diffusion. We were able to dissect in real time the burst-like assembly and disassembly of the receptor nanoclusters, and their duration, size and density. Moreover, a correlation could be established between the behaviour at the individual trajectory level and the heterogeneous motional behaviour at the ensemble level: we found that the nanoscale dynamics of the single-trajectories dictate the population dynamics at the mesoscopic level, and that cholesterol fine tunes free walk diffusion and the stability of confinement zones.

## Results

### Heterogeneity of nAChR mobility

**Figure 1:**
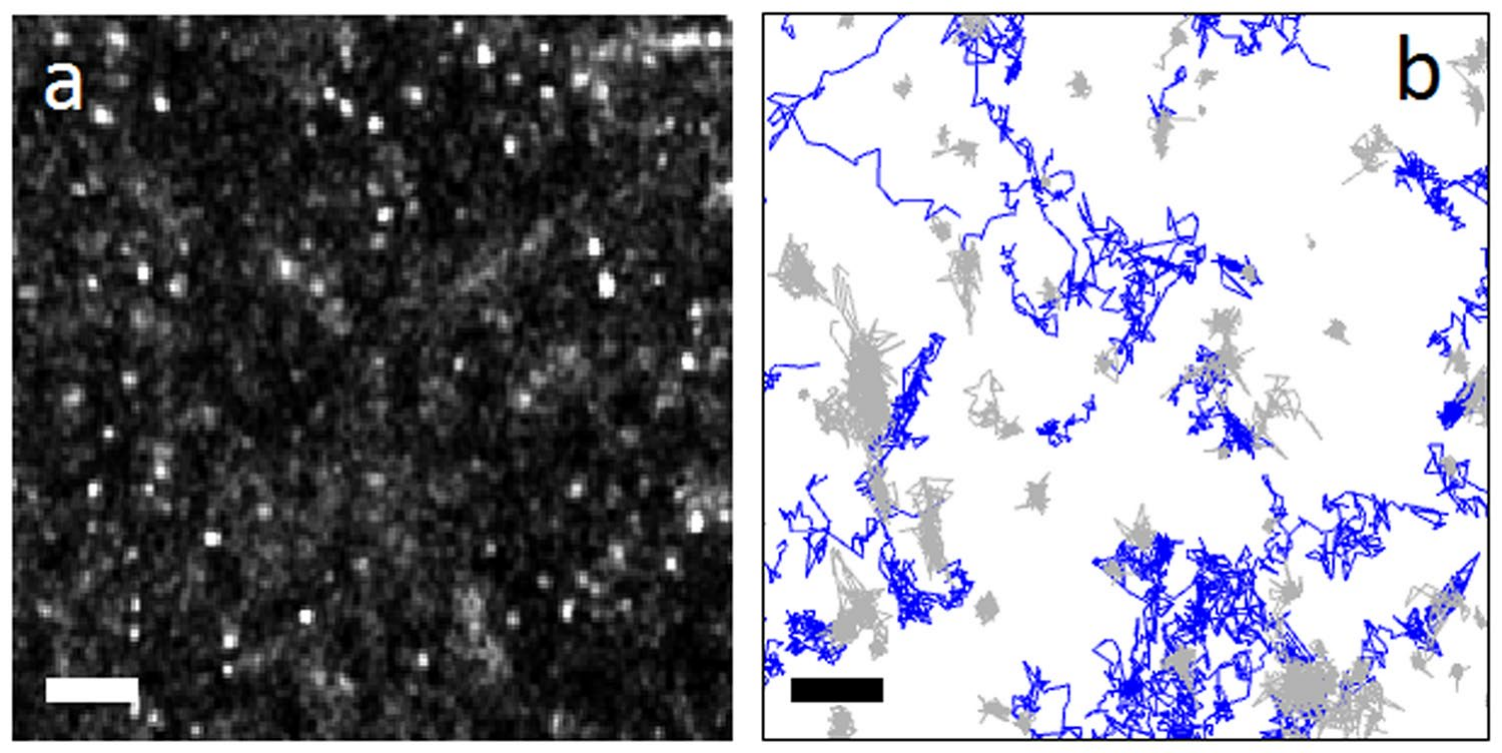
Raw localizations and nAChR trajectories. (**a**) A 3.8 × 3.8 μm Gaussian-smoothened projection image of a CHO-K1/A5 cell stained with AlexaFluor^555^-BTX and subjected to cholesterol enrichment (CDx-Chol), showing the identified and validated localizations. (**b**) Mobile (blue) and immobile (grey) nAChR trajectories as defined in ref.^11^. Scale bar: 500 nm.

Live CHO-K1/A5 cells labelled with fluorescent Alexa Fluor^555^ α-bungarotoxin (BTX) were imaged in STORM buffer at a rate of 100 fps. Regions of interest (10 × 10 μm) were selected from an average of 10 cells for each experimental condition. Upon reconstruction of the STORM images, the validated localizations were found to be distributed nonuniformly across the cell surface in the form of puncta of varying fluorescence intensities (Fig. 1a). Supplementary Fig. 1 shows the precision of the localizations. A representative example of single-molecule BTX-labelled nAChR trajectories with more than 50 steps (some up to 1600 steps) recorded from ventral surface membrane areas of live CHO-K1/A5 cells is shown in Fig. 1b. Visual inspection of the tracks already reveals the heterogeneity of the trajectories: all sets of fluorescent-labelled samples exhibit free walks of variable stretch and regions of apparent confinement in restricted domains. Since the weight of truly immobile particles could affect the outcome of the analysis of the mobile population, this observation called for a distinction to be made between confined but mobile receptors and truly immobile ones, and to exclude the latter.

In order to exclude the immobile molecules from the analysis, we next applied a recently introduced sorting procedure^11^ which combines the radius of gyration and the mean displacements of the particles (see Supplementary Fig. 2b). The normalized ratio ($\sqrt{\pi /2}({R}_{g}/\langle |{\rm{\Delta }}r|\rangle )$ obtained from the experiments with *fixed* cells is a constant independent of the localization error or the threshold diffusion coefficient (*D*_*th*_)^11^. Using this approach, we calculated the threshold value to exclude immobile molecules in our *live* cell experiments (Supplementary Fig. 2c). Threshold values of 1.5–2.1 (95% confidence) were obtained by pooling data from different cells in two independent sets of experiments, and the conservative value of 2.1 was chosen. The pooled mean values of immobile receptors varied between 50 and 65% (Supplementary Fig. 2). The immobile nAChR trajectories were excluded from further analysis.

**Figure 2:**
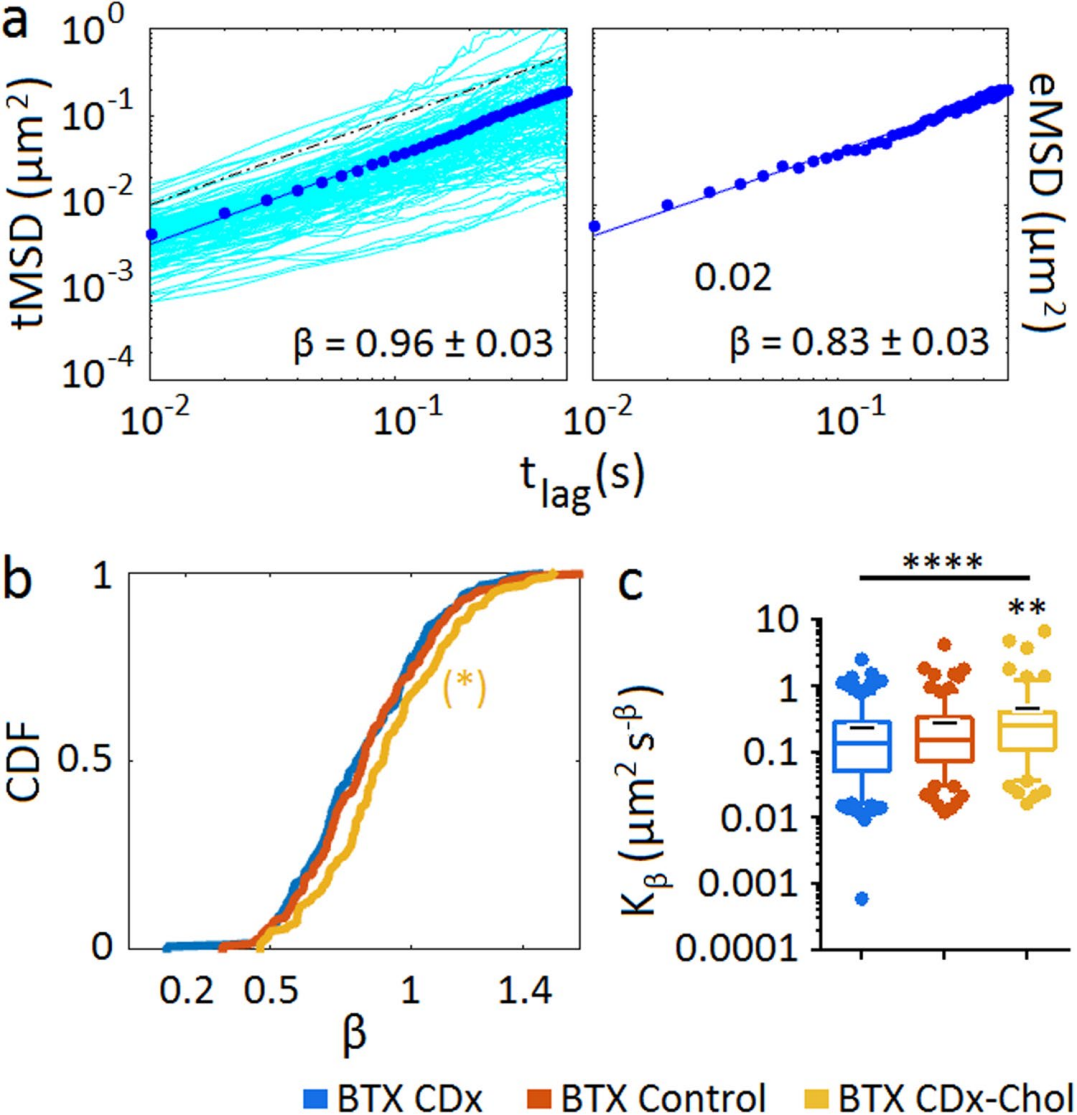
Time-averaged (tMSD), ensemble-averaged MSD (eMSD), cumulative distribution function of anomalous exponent β and generalized diffusion coefficient *K*_*β*_ distribution of the total populations of nAChR trajectories, under control and cholesterol-modifying conditions. (**a**) The top-left panel is the log-log plot of the tMSD corresponding to control nAChRs including all the individual trajectories having a goodness of fit better than 0.9 (see Supplementary Material). The dots correspond to the average tMSD and the solid line is the fit to the log-log transformed data. The exponent β values ±95% confidence interval are indicated in each case. The dashed line is a visual aid that scales linearly with the log of time. The top-right panel shows the linear fitting (blue full line) to the log-log transformed eMSD (solid points). The fits scale as *β*log(*t*_*lag*_), rendering the power (anomalous) exponent β. (**b**) Cumulative density distribution function (CDF) of the anomalous exponent β. (**c**) Effect of cholesterol depletion (CDx) or enrichment (CDx-Chol) on the generalized diffusion coefficient, *K*_*β*_. Whiskers in box plots correspond to 95% confidence intervals; the limits indicate 75% confidence intervals; the black – symbols indicate the mean and the horizontal lines the median in each case. The dots outside the confidence intervals are outliers. Statistics: (*)p < 0.05, (**)p < 0.01 and (****)p < 0.0001.

To characterize the heterogeneous diffusional behaviour of the nAChR mobile molecules upon removal of stationary ones, we first analysed the mean-square displacements (MSDs). The MSD curves can be approximated as $\sim {t}_{lag}^{\beta }$, as explicitly stated in Eq. 4 in Supplementary Material. By linearly fitting the log-log transformed MSD curves we obtained the power-law scaling, i.e. the anomalous exponent β. Figure 2b shows the cumulative density function (CDF) of the anomalous exponent β for the entire (unsorted) populations of control samples and their corresponding curves upon cholesterol depletion or enrichment. The criterion for the choice of β is the same as that adopted in ref.^12^, i.e. the use of a non-dimensional parameter. The CDF graphs show that cholesterol enrichment increases diffusivity, albeit to a small extent (β values were 0.92 ± 0.02, 0.96 ± 0.03 and 1.02 ± 0.02 for cholesterol-depletion, control, and cholesterol-enrichment conditions, respectively). The wide span covered by the β exponent, from ca. 0.3 to 1.6, leads us to consider the possibility that the high heterogeneity of the nAChR mobilities could be a consequence of ergodicity breaking.

### nAChR trajectories display weak ergodicity breaking

To evaluate whether the observed anomalous nature of nAChR diffusion was associated with ergodicity breaking, we compared the time-averaged and ensemble-average MSDs for the whole population of trajectories. The tMSD plots include all the individual trajectories and the fit corresponds to the linear regression to the average of the individual time-averaged trajectories (in log scale), whereas the eMSD distribution results from averaging the square displacement of all trajectories occurring at a given interval (Fig. 2a). The span of the tMSD is broad in all cases, indicating that they do not self-average. The values of β are indicated in the two plots, and a comparison of all the experimental conditions is given in Table 1. In ergodic systems, tMSD and eMSD converge to similar values for large numbers of diffusing molecules and long enough times. Here, control samples exhibit weak ergodicity breaking (Table 1). The MSD data provide additional information on the changes induced by cholesterol on nAChR mobility (Supplementary Fig. 3), especially in the case of cholesterol-enriched samples, where the ensemble diffusivity was markedly increased (Fig. 2b). Figure 2c shows the corresponding generalized diffusion coefficient *K*_*β*_ of nAChR single molecules under control and cholesterol-modifying conditions, which also exhibited a broad distribution covering more than two decades (0.23 ± 0.04 μm^2^ s^−β^, 0.26 ± 0.06 μm^2^ s^−β^, and 0.43 ± 0.14 μm^2^ s^−β^, for cholesterol depletion, control, and cholesterol enrichment conditions, respectively). Highly significant statistical differences were observed between the *K*_*β*_ values of cholesterol-enriched and control samples (p < 0.0001), mimicking the trends observed with the exponent β.

**Table 1:** Ergodicity analysis of nAChR trajectories applied to the entire population of BTX-labelled nAChR trajectories under different cholesterol-modifying conditions*.

	CDx	Control	CDx-Chol
tMSD	0.92 ± 0.02	0.96 ± 0.03	1.02 ± 0.02
eMSD	0.90 ± 0.02	0.83 ± 0.03	0.96 ± 0.04

*The anomalous exponent β corresponds in both cases to the slope obtained from the linear fitting to the log-log plot of the single molecule average tMSD and the corresponding eMSD, i.e. the average across the ensemble of trajectories as described under Material and Methods in the Supplementary Material. Mean ± 95% confidence intervals are shown.

### Underlying physical mechanisms: Escape (waiting) time and turning angle probability analyses

To investigate the possible underlying physical substrate of the observed nAChR subdiffusive behaviour, we resorted to analysis of the distribution of two variables: the probabilities of the escape times and of the turning angles.

**Figure 3:**
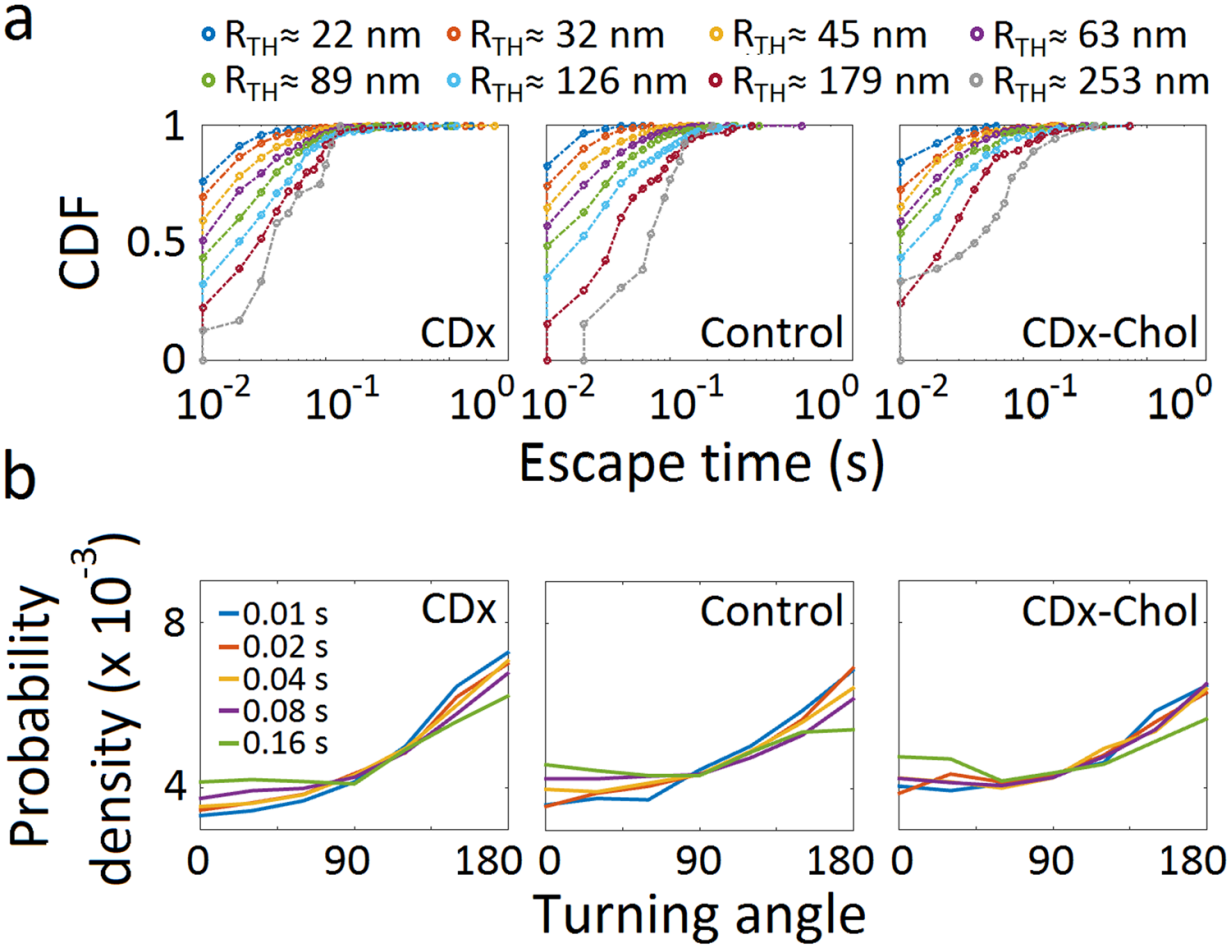
Escape (waiting) time distributions and turning angle probability densities of nAChRs. (**a**) Cumulative density (distribution) function of the escape (waiting) times, corresponding to increasing radii *R*_*TH*_ from 22 nm to 253 nm. (**b**) Probability densities for the colour-coded t_lag_ of increasing durations (10 to 160 ms). The probability density is normalized such that the integral of the curve is equal to unity.

Continuous time random walk (CTRW) is a non-ergodic anomalous diffusion model consisting of random walks with transient immobilization, with a dwell-time probability density that scales as ~t^−(*β*+1)^. If *β* ≤ 1, the mean dwell-time diverges, and the experimental time window cannot reach the characteristic time of the system, leading to ergodicity breaking. The escape (waiting) time is the interval during which a trajectory remains within a given radius *R*_*TH*_ and can be used to test whether the CTRW model explains the experimental data. If the long-time dynamics of trajectories is dominated by the immobilization events, then the waiting time distribution should not depend on the escape radii. We quantified the duration of the events in which the molecules’ trajectories remained within circular areas of increasing radii *R*_*TH*_^13,14^. The distribution of waiting times showed a clear dependence on escape radius between ~20 to 250 nm (Fig. 3a), strongly suggesting that the ergodicity breaking observed under control conditions (see also Table 1) cannot be satisfactorily accounted for by the CTRW model.

We next analysed the directional changes in individual nAChR trajectories using the relative angles distended by the molecules along their walk, a parameter which can help distinguish among different types of subdiffusive mechanisms^15,16^. The turning angle distribution tests for correlations in the particle displacements (see graphical explanation in Supplementary Fig. 5). When applied to the total population, the probability of the turning angles was found to increase gradually from 60–90° onwards to peak at *θ* = 180° for *all* experimental conditions (Fig. 3b), a clear indication of anticorrelated steps. A time-lag dependence was apparent in the directional changes of the control and cholesterol-depleted conditions, suggesting diffusion in a meshwork, a condition compatible with permeable fence models^15^.

### Classification of trajectories into diffusivity-based subgroups

**Figure 4:**
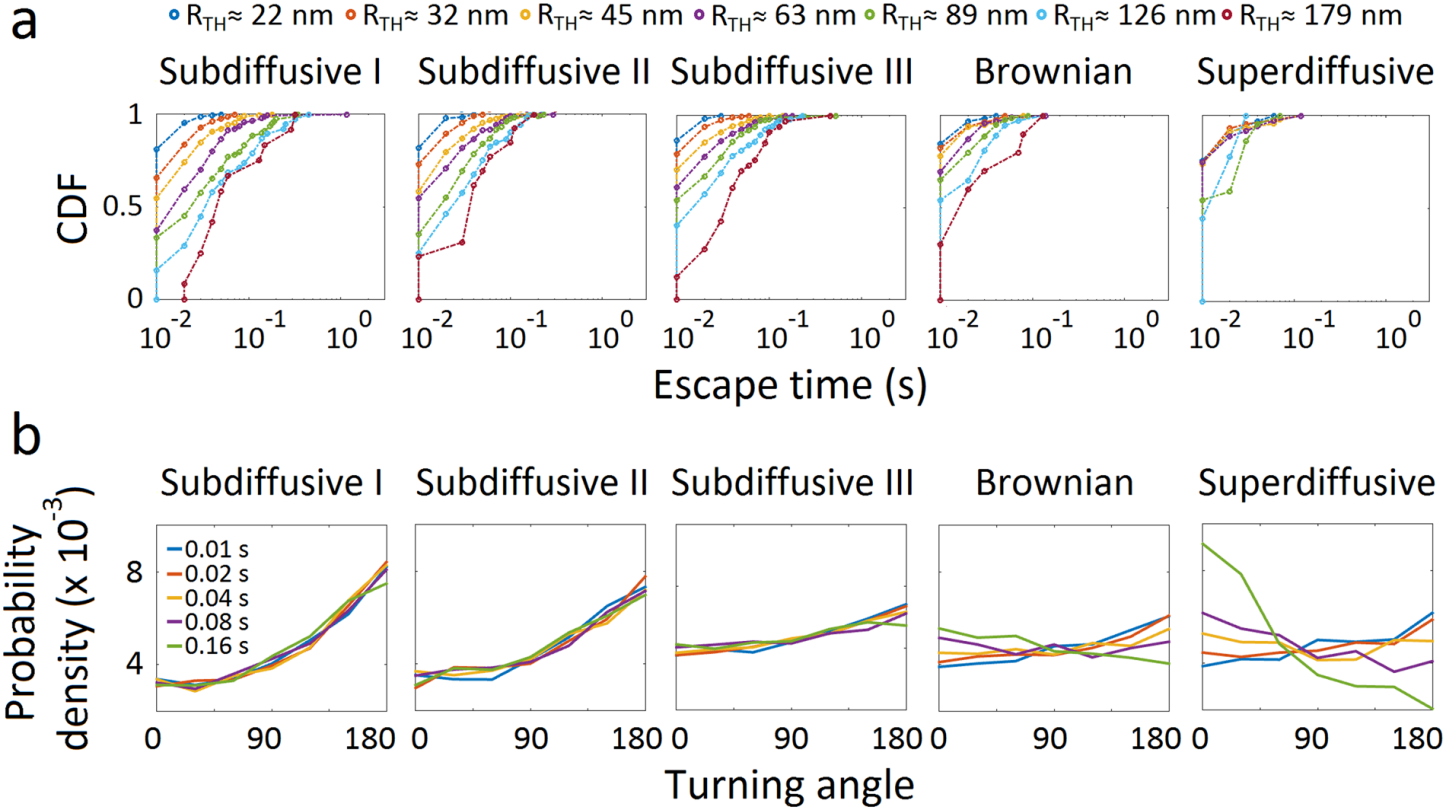
Analysis of diffusivity-based trajectories. (**a**) Cumulative density function (CDF) of escape (waiting) times corresponding to increasing radii *R*_*TH*_ for the diffusivity-based subpopulations under control conditions. (**b**) Turning angle probability density distribution of each subpopulation (control condition). Turning angles correspond to increasing time lags indicated by the colour code, from 10 ms (light blue) to 160 ms (green). The probability density is normalized such that the integral of the curve is equal to unity.

We next separated nAChR trajectories according to their diffusivities. Based on the power exponent β, trajectories can be grouped into subdiffusive I (*β* < 0.5), subdiffusive II (0.5 < *β* ≤ 0.7), subdiffusive III (0.7 < *β* ≤ 0.9), Brownian (0.9 < *β* ≤ 1.1), and superdiffusive (*β* > 1.1) subpopulations. Using this classification, several properties of the different motional motifs could be identified, which were otherwise hidden in the unsorted population (Fig. 3). Whereas the escape (waiting) time of the subpopulations is like that of the ensemble population of trajectories (Fig. 4a), the turning angle probability distribution shows distinct features: it increases from 60–90° onwards to peak at *θ* = 180° for subdiffusive subpopulations, with a progressively decreasing slope from the subdiffusive I to the subdiffusive III subpopulations; in contrast, the Brownian trajectories exhibit a nearly flat and uniform turning angle distribution with anticorrelated steps at short time lags and correlated steps at long time lags (Fig. 4b). The same tendency is observed in the superdiffusive subpopulation, which exhibits a more pronounced step correlation (peak at *θ* = 0°) at long time lags (40 ms to 160 ms). Similar trends in angular dependence are observed under cholesterol-modifying conditions (see Supplementary Fig. 6).

### Microscopic heterogeneity: transient confinement nanodomains within individual trajectories and differences between subdiffusive states

**Figure 5:**
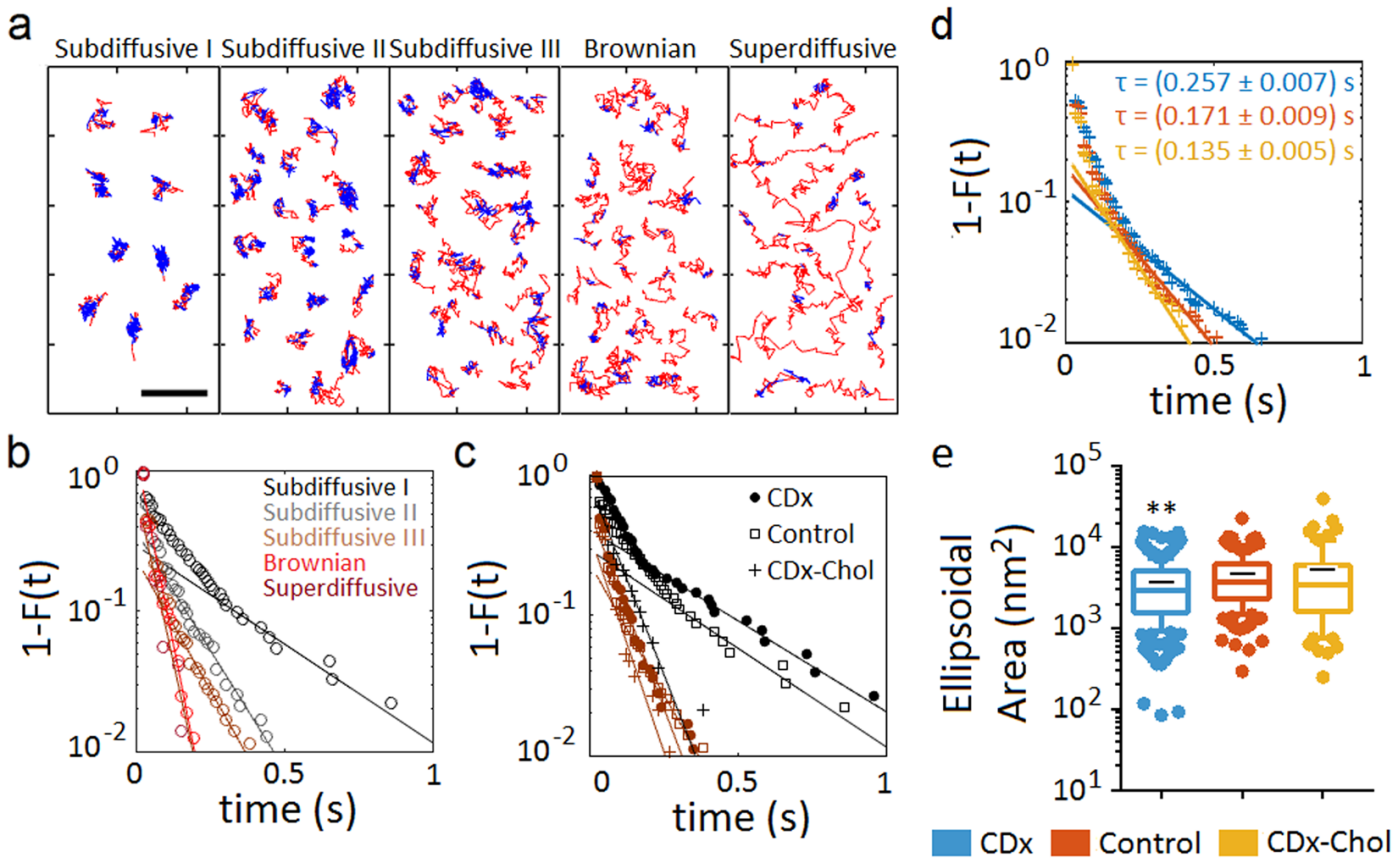
Microscopic heterogeneity: transient confinement in nanodomains within individual trajectories and differences between subdiffusive states. (**a**) Recurrence analysis of individual trajectories^41^ identifies the unconfined diffusing portion of each individual trajectory (red) and transient confinement sojourns (blue) within the trajectories under control conditions. Notice the decreasing immobilization (confinement) from the subdiffusive I to the superdiffusive subpopulation. Bar: 1 µm. (**b**) Complementary cumulative distribution function of the residence times in the confined state of each subpopulation (control conditions). The tails of the curves were fitted with exponential decay functions, from which the decay times were obtained (listed in Supplementary Table 2). (**c**) The same as in (**b**) but showing the effect of cholesterol modification on the subdiffusive I and III subpopulation (same colour code as in (**b**)). (**d**) The same as in (**c**) but for the total population of nAChRs after cholesterol enrichment and depletion. (**e**) Ellipsoidal area of the trajectories’ portion in the confined state, obtained by fitting the confinement domain in the trajectory with an elliptic surface. Whiskers in box plots correspond to 95% confidence intervals; the limits indicate 75% confidence intervals; the black − symbols indicate the mean and the horizontal lines the median in each case. The dots outside the confidence intervals are outliers. (**)p < 0.01.

**Figure 6:**
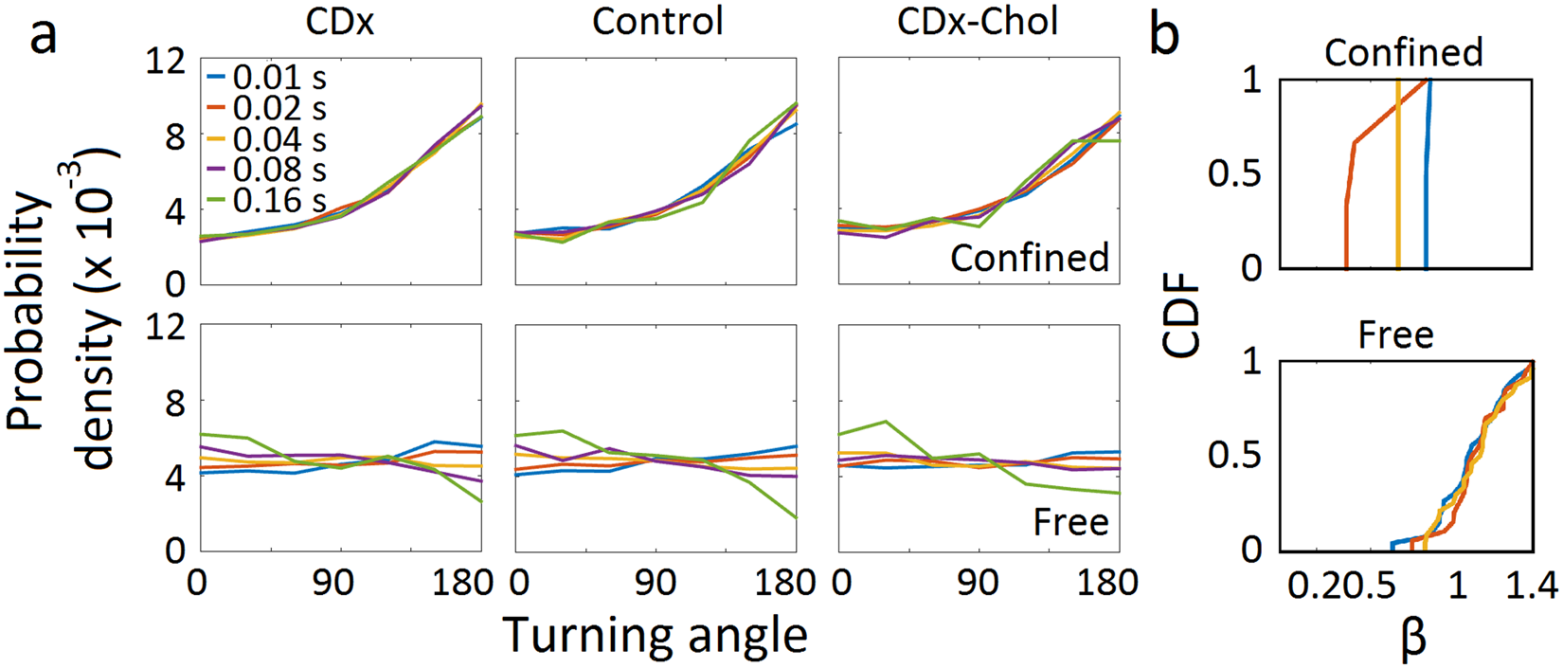
Dynamic characterization of the transiently confined and unconfined portions of the individual trajectories in control and under cholesterol-modifying conditions. (**a**) Turning angle distribution corresponding to increasing lag times (indicated in the top left panel), from 1 frame (10 ms, blue) to 16 frames (160 ms, green) for the confined portions and the unconfined, free portions of the trajectories having at least 50 steps. The probability density is normalized such that the integral of the curve is equal to unity. (**b**) Distribution of the anomalous exponent β for free and confined portions in the ensemble, total population of nAChRs under cholesterol-depletion and -enrichment.

We next focused on the behaviour of individual trajectories. To this end, we applied a series of recently introduced analytical tools^17^ to scrutinize in step-by-step detail the single-molecule tracks. One such tool (recurrence analysis) examines the recurrence of individual trajectories, i.e. whether a molecule stops at the same location that it has previously visited at the membrane surface. This analysis discriminates between different dynamics and characterizes the shape of the confinement areas, rendering the signature motional behaviour at the single-molecule level. Remarkably, essentially all trajectories, independently of their diffusional modality, exhibit an intermittent alternation between unconfined free-diffusing portions and sections of the track in which the molecule is confined for varying intervals within small (nanometre-sized) areas (Fig. 5a). The ellipsoidal areas covered by the confinement sojourns have an average major semi-axis of ca. 40 nm (39 ± 2 nm, 45 ± 3 nm and 46 ± 6 nm for cholesterol-depletion, control, and cholesterol-enrichment conditions, respectively) and an eccentricity of ~0.6. The ellipsoidal surfaces are equivalent to circular areas with radii of 32 ± 2 nm, 36 ± 2 nm and 37 ± 4 nm for cholesterol depletion, control, and cholesterol enrichment conditions, respectively. The longest residence times in the confinement sojourns (Fig. 5b) correspond to the subdiffusive subpopulation I and II, followed by the subdiffusive III, Brownian and superdiffusive groups, in that order. Changes in cholesterol levels modulate the lifetime of the confinement sojourns, more markedly for the less diffusive subpopulations (Fig. 5c,d). Also, the confinement areas diminish in size upon cholesterol depletion (Fig. 5e). The proportion of the trajectories spent in the confined state is ~30%, changing upon cholesterol modification (Supplementary Fig. 9b). Furthermore, marked differences are observed in the % of the confined state per single-molecule track between the different diffusivity-based subpopulations, especially for the subdiffusive ones (Supplementary Fig. 9a), an observation that led us to analyse them separately. Analogously, whereas the cumulative distribution function β corresponding to the free portions of the unsorted, averaged population varied from Brownian to nearly superdiffusive (β = 1.07 ± 0.08, 1.10 ± 0.08 and 1.10 ± 0.08 for cholesterol-depletion, control, and cholesterol-enrichment conditions, respectively), the subdiffusive behaviour is only apparent in the confined portions (Fig. 6b), suggesting that the confinement sojourns in nanodomains are the ones that determine the predominant subdiffusive behaviour of the nAChR population. The ability to dissect individual trajectories into confined and free regions (Fig. 5) additionally allowed us to apply turning angle analysis to the two sections separately. The confined regions show a clear anticorrelation (peak at *θ* = 180°) and time invariance (i.e. independent of time lag) for all experimental conditions (Fig. 6a). In contrast, the free (unconfined) portions appear to conform to the behaviour typically observed for Brownian diffusion, i.e. an angle-independent, nearly flat uniform distribution, but exhibit time lag dependence, Brownian-like flatness at short times and are positively correlated, with a peak at *θ* = 0° at long times. Clear statistical differences are observed between the anomalous exponent β of free and confined trajectories (Fig. 6b; p < 0.01 and p < 0.0001 for control and cholesterol-enriched conditions, respectively).

### Disclosure of nAChR nanocluster formation/disassembly dynamics in real time

**Figure 7:**
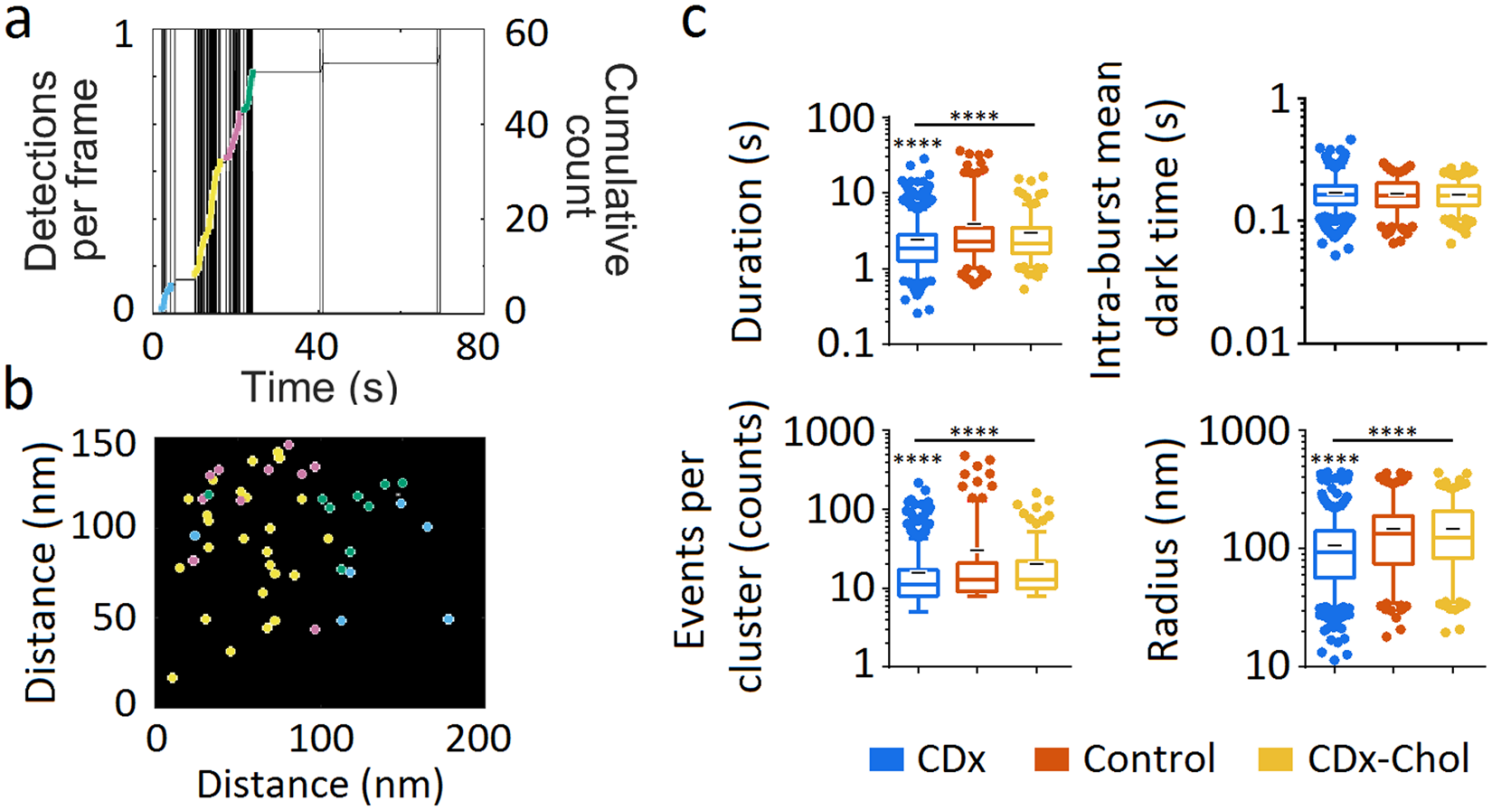
Real time follow-up of nAChR dynamic nanoclusters under cholesterol depletion and enrichment. (**a**) Using the live-cell single-molecule localizations validated with ThunderSTORM as the input data, the qSR software was applied to generate the time-dependent plot: The bursts of individual spikes identify the formation of dynamic nanoclusters; the cumulative count traces (ascending continuous coloured line) superimposed on the spikes delimit each (nano)cluster in a different colour. The horizontal plateau sections of the trace correspond to the nanocluster breakages^19^. (**b**) A 150 × 200 nm region showing single-molecule cluster localizations (corresponding to the cumulative traces shown with the same colour in (**a**)). (**c**) nAChR nanocluster metrics (log scale): duration, intra-burst mean dark time, i.e. periods without localizations within a cluster, events per cluster, and RMS radii of clusters under control and cholesterol modifying conditions^20^. Whiskers in box plots correspond to 95% confidence intervals; the limits indicate 75% confidence intervals; the black  negative symbols indicate the mean and the horizontal lines the median in each case. The dots outside the confidence intervals are outliers. Statistics: (****)p < 0.0001.

We next employed the qSR algorithms developed by Cisse and coworkers^18^ to follow nAChR nanocluster dynamics in real time. As shown in Fig. 7a, bursts of activity in the form of vertical spikes (ascending coloured cumulative count trace), and periods of “inactivity” (grey horizontal lines in the cumulative count trace), conform the signature of cluster formation/dissociation^19,20^. The spatial arrangement of the single-molecule localizations belonging to each dynamic nanocluster in the confinement regions of the traces is shown in Fig. 7b. Cholesterol modification induces marked statistical differences in the nanocluster metrics: Nanoclusters exhibited RMS radii of 107 ± 4 nm, 149 ± 13 nm and 149 ± 13 nm for cholesterol depletion, control, and cholesterol enrichment conditions, respectively; lifetimes in the order of seconds (2.45 ± 0.14 s, 3.90 ± 0.77 s and 2.96 ± 0.32 s for cholesterol depletion, control, and cholesterol enrichment conditions, respectively); and tens of particles per cluster (16 ± 1, 30 ± 8 and 20 ± 3 particles for cholesterol depletion, control, and cholesterol enrichment conditions, respectively) (Fig. 7c). No statistical differences were observed between control and cholesterol-modified samples in the intra-burst (“dark”) periods lasting ~0.17 s.

## Discussion

The ultimate aim of this work is to investigate how variations in synapse cholesterol content influence nAChR mobility. We have chosen a model mammalian heterologous clonal cell line produced in our laboratory which robustly expresses adult-type nAChR^21^ to test in a straightforward manner a focused set of objectives of this aim, technically inaccessible in the intact synapse, using state of the art high-density single molecule tracking, single-molecule localization-based superresolution microscopy, and computational analytical tools.

### Heterogeneous behaviour of the ensemble population of nAChR trajectories

The first general observation stemming from our multi-pronged analyses concerns the complexity and heterogeneity of nAChR dynamics. The cell-surface membrane of mammalian cells is a complex environment from both structural and functional viewpoints. It is in fact surprising that some membrane proteins manage to diffuse by thermally-driven, viscosity-dependent, uncorrelated Brownian motion. Deviations from Brownian motion, i.e. anomalous diffusion, in such a crowded and heterogeneous milieu is more often than not the rule^22–24^, as has also been observed with the nAChR^25–27^.

Recently He and coworkers^28^ followed the mobility of quantum-dot labelled nAChRs in *Xenopus* muscle cells in culture. The nAChR did not obey the Gaussian statistics characteristic of Brownian diffusion. They interpreted these observations as resulting from dynamic heterogeneity, and proposed a variant of Kusumi’s picket-fence model^29,30^ which they termed “dynamic picket-fence model”. Kusumi’s original model purports the existence of “corrals” formed by sub-membrane actin filament network “fences” converging onto “pickets” of actin-binding proteins. Pickets and fences act as percolation barriers that diffusing proteins must “hop over” to dodge the corrals. In the present experiments, we observe confinement areas with a normalized radius of ~36 nm in control samples; actin corral immobilization operates on membrane areas in this spatial scale^30–32^. He and coworkers^28^ also argued that immobile nAChR tracks may contribute to the non-ergodic MSDs, but in the present work we have specifically addressed and discarded this possibility using recently established criteria^11^ to exclude immobile trajectories.

### Physical mechanisms behind the anomalous nAChR dynamics

Ergodicity lies at the core of statistical mechanics; the convergence of the temporal and the ensemble averages is known as the ergodic hypothesis (see review in ref.^33^). Diffusion processes that deviate from Brownian motion are considered anomalous, and their propagators may or may not be Gaussian^14,34^. However, non-ergodic systems can be conformed to nearly Gaussian, probably due to the probe only sampling a local region during the time scale of the observation^35^. This is particularly important for some models having slow convergence, such as the obstructed diffusion (OD) model with obstacle concentrations close to criticality^33^. Our experiments cover a limited temporal window, within which the comparison of the time-averaged MSD with the ensemble-averaged MSD revealed the presence of both ergodic and non-ergodic behaviours at the ensemble level (Fig. 2 and Table 1). We therefore attempted to discriminate between plausible physical models accounting for such complexity.

The distribution of relative angles of motion between successive time intervals of a trajectory provides information beyond that afforded by MSD analyses^15,16^. A preferred angle of 0° indicates that the moving particle travels in the same direction as the previous step; in contrast, a preferred angle *θ* = 180° specifies that the molecule walks in the opposite direction to the previous step. The latter appears to be the case with the trajectories of the total population (Fig. 3) as well as with all subdiffusive trajectories (Fig. 4 and Supplementary Fig. 6), indicating that nAChR molecules turn back rather than continue their motion in a defined direction, a fingerprint of subdiffusive random walks with anticorrelated increments^15,33^. Could this be accounted for by viscoelastic properties of the membrane or could it be the result of interactions with physical obstacles? Turning angle analysis allowed us to discriminate between these two scenarios. The fractional Brownian motion (fBM) model (a generalization of Brownian motion with power-law correlated displacements and a Gaussian propagator) describes diffusion in viscoelastic fluids, whereas the OD model refers to particles hindered by near-immobile obstacles. Unlike Brownian motion, in the fBM model particles revisit previously visited locations^36^ and exhibit a time-dependent diffusion coefficient^17,33,37^. Krapf and coworkers^15^ have pointed out that although both the fBM and the OD models display turning angle distributions peaking at *θ* = 180°, fBM exhibits a gradual increase between 45° to 135° and a plateau at higher angles, whereas the OD model shows a continuous increment between 90° and 180°. The subdiffusive nAChR trajectories exhibit the latter signature (Figs 3 and 4 and Supplementary Fig. 6), suggesting the fBM model should be discarded in favour of the OD model and indicating the presence of obstacles impeding or delaying nAChR motion. The anticorrelated steps are time lag-dependent, a feature consistent with diffusion in a meshwork^15^ and the picket-fence model (see recent update in ref.^38^). In comparison with the numerical simulations of ref.^15^ the steepness of the increase in the subdiffusive type I and II subpopulations suggests obstacle concentrations close to criticality, and the declining slope from the subdiffusive I towards the Brownian subpopulation submits to either (i) a decreasing gradient of obstacle concentration^15^ or the possibility that (ii) the ratio of free/confined regions of the individual trajectories increases accordingly. Characteristic obstacles for membrane protein lateral diffusion are protein self-aggregation, pickets of other immobile or slowly moving proteins, sub-membrane actin corrals, or liquid-ordered (Lo) lipid heterogeneities (“rafts”). Any of these obstacles, or mixtures thereof, add complexity to the mechanism of transient immobilization and can give rise to apparent non-ergodic behaviour^39^, particularly because they may act on different spatial and time scales. This may be the case with the nAChR trajectories that exhibit weak ergodicity breaking.

Examination of the duration of the events in which the confined region of the trajectories remained within circular areas of increasing radii *R*_*TH*_^13,14^ provided the opportunity to test the CTRW model^22–24^. In this hypothesis, molecules move randomly, with waiting times also random, with a probability *τ*^−(*β*+1)^, and average tMSD linearly scaling with lag time. CTRW is a non-ergodic model of subdiffusion associated with binding to stationary components such as the actin meshwork^14^, molecular crowding, protein-protein interactions or lipid domains, all of which may give rise to transient immobilization. Instead, a higher, close-to-criticality obstacle concentration could explain the phenomenon, resulting in turn from e.g. stabilization of the actin cytoskeleton^40^ with convergence times beyond the temporal window explored in the present work.

### Microscopic heterogeneity: transient confinement nanodomains interrupt individual trajectories

Application of a series of analytical tools recently introduced by Krapf and his group to inspect individual trajectories in detail^41^ led us to disclose the occurrence of pauses -remarkably in *all* single nAChR trajectories, including Brownian and, less frequently, even superdiffusive trajectories. The latter may result from active processes associated with molecular motors/the submembrane cytoskeleton or have their origin in clustering/de-clustering events like those observed with submicron tracers in micellar solutions. The mobile tracks were interrupted in all cases by sojourns of variable duration, whereby receptors were transiently confined within nm-sized domains of ca. 36 nm. Other SPT studies reported that 20% of nAChRs in muscle myoblasts showed restricted diffusion in small domains having similar dimensions^26^. We found that the less diffusive subpopulations spent the longest stopovers in the confined state; the confined sojourns of Brownian trajectories had shorter durations, with superdiffusive trajectories displaying the shortest confined lifetimes (Fig. 5, Supplementary Fig. 7 and Supplementary Table 2). Thus, receptor dynamics appear to be governed by the probability of encounter and the time spent in confined and free-diffusing regions of the trajectory. Modification of the cholesterol levels changed the characteristic lifetime and the areas of the confined sojourns (Fig. 5d,e).

### Modulation of nAChR diffusional dynamics: synergistic effects of cholesterol and receptor self-crowding and trapping in confined regions

Cholesterol possibly affects nAChR diffusivity through two non-exclusive and complementary mechanisms: (i) via the physical state of the bulk bilayer, overwhelmingly determined by the chemical composition of the constituent lipids, and (ii) through changes in the abundance and/or size of cholesterol/sphingolipid-rich nanodomains. Mechanism (ii) appears to influence mainly the second-long kinetics of formation and disassembly of nAChR nanoaggregates which we could follow in real time (Fig. 7). Additionally, the turning angle behaviour of the nAChR walks with anticorrelated steps (Figs 3–4 and 6), can be satisfactorily accounted for by the occurrence of cholesterol/sphingolipid-rich Lo domains acting as lateral heterogeneities in the plane of the membrane, i.e. obstacles that nAChRs have to circumvent in a permeable fence scenario, in accordance with the notion that heterogeneity of the diffusing bodies’ environment has a strong impact on mobility^35^. The possibility that nanometre-sized Lo domains have a role (direct or indirect) in inducing confinement finds independent experimental support. When reconstituted in a sphingomyelin-cholesterol-POPC (1:1:1) model system, purified nAChR from *Torpedo* does not exhibit preference for partitioning in Lo domains^42^. However, inclusion of sphingomyelin molecular species that generate bilayer asymmetry by enriching the sphingolipid content of the outer leaflet appear to favour the inclusion and enrichment of the nAChR in Lo domains^43^. Thus, nAChRs can inhabit/be excluded from Lo domains (“rafts”) depending on the composition of the local lipid microenvironment, and this, in turn, facilitates receptor self-aggregation. This is particularly relevant to the function of the cholinergic synapse: in addition to the quite high concentration of cholesterol compared to other membrane lipids in the neuromuscular junction or in the *Torpedo* electromotor synapse^44^, one outstanding feature of these two synapses is the extraordinarily high density of nAChR protein^45^, a feature shared with the CHO-K1/A5 cell line^10^ studied here albeit to a less pronounced degree. Macromolecular crowding in cell membranes is known to produce deviations from Brownian diffusion (reviewed in ref.^46^). In our hypothesis, the cholesterol-rich Lo domains could hinder or reduce diffusion and concentrate sluggish or immobile nAChRs. We excluded immobile receptors from the diffusional analysis, but one must bear in mind that these static macromolecules constitute nonetheless a major proportion of the total population (and as such are included in the clustering analysis). Recently, cholesterol-dependent structures coined “cages” were reported in the CHO line, parent cells of our CHO-K1/A5 line^47^. Cages are larger and longer-lived than the purported size of some “raft” domains and are stabilized within even larger and longer-lived actin corrals. Cholesterol depletion destabilizes these diffusional barriers, as we observe with the nanoclusters in the present work (Fig. 7). The confinement nanodomains that we observe (~36 nm radius) are similar to others recently disclosed in static imaging of cholesterol-dependent nanodomains, such as those harbouring dopamine receptors (~35 nm radius^48^), or GPI-anchored protein raft-born nanodomains (~40 nm^38^).

The question arose as to whether the different diffusional regimes are due to different concentrations of obstacles or to the relative time spent in confined/free zones. When we dissected the individual trajectories into confined and free regions, it became clear that the turning angles are essentially the same in each region, indicating that the nAChR in confinement regions encounters similarly high obstacle concentrations -close to criticality- or a similar probability of permeation under all experimental conditions (Fig. 6), affecting *all* diffusive regimes (Supplementary Fig. 8) in marked contrast to the behaviour of the free diffusing regions (Fig. 6 and Supplementary Fig. 8). This is a key observation: the time spent in confined sojourns relative to that in free walks determines the resulting motional regime and the breadth of the macroscopic heterogeneity in the ensemble population.

In addition to the transient and reversible nanoscale confinement sojourns within single nAChR trajectories, lasting tens of milliseconds along the individual walks, we disclosed another dynamic process, occurring in the time window of seconds and of a greater spatial extension. This much slower dynamic process likely corresponds to the assembly/residence/disassembly of nanoclusters within confinement areas. We observed statistically significant differences in the RMS radius and number of events in these nm-sized nanoclusters, as well as in the lifetime of the nanocluster assembly period, following changes in cholesterol content (Fig. 7). Thus, the alternating free diffusion + confined diffusion at the single-molecule level is reflected at the mesoscopic level in the “social” behaviour of the receptor, i.e. its tendency to reversibly self-associate in a cholesterol-dependent manner in supramolecular aggregates at the cell surface. The signature link between the two processes is their cholesterol dependence, modulating diffusion in the bulk bilayer and stabilizing nanodomains in the confinement regions.

## Electronic supplementary material

Supplementary Material
