# Supplementary material for: Cholesterol modulates acetylcholine receptor diffusion by tuning confinement sojourns and nanocluster stability
Source: Sci Rep. 2018 Aug 10;8:11974. doi: 10.1038/s41598-018-30384-y (PMC6086833; doi:10.1038/s41598-018-30384-y)
Supplement: Supplementary file 1 — Supplementary Material [file 41598_2018_30384_MOESM1_ESM.docx]

**Cholesterol modulates acetylcholine receptor diffusion by**

**tuning confinement sojourns and nanocluster stability**

**Alejo Mosqueira, Pablo A. Camino and Francisco J. Barrantes^*^**

Laboratory of Molecular Neurobiology, Institute for Biomedical Research,

UCA–CONICET, Av. Alicia Moreau de Justo 1600, C1107AFF Buenos Aires, Argentina.

**Supplementary Material**

**Materials**

Methyl-β-cyclodextrin (CDx), catalase, glucose oxidase, β-mercaptoethanol, polyvinylalcohol (PVA, 25,000 MW, prod. No. 184632) and the monoclonal antibody mAb35 (product M-217) against the extracellular moiety of the nAChR α-subunit were purchased from Sigma Chem. Co. (St. Louis, MO). Native, unlabelled α-bungarotoxin (BTX) and Alexa Fluor-labelled BTX (Alexa Fluor^555^-BTX), Alexa Fluor^555^- or Texas Red-labelled anti-IgG secondary antibodies were purchased from Invitrogen Argentina.

**Cell culture**

CHO-K1/A5 cells were grown in Ham’s F12 medium supplemented with 10% foetal bovine serum for 2-3 days at 37°C (ref. [^1^](#_ENREF_1)) before experiments.

**Acute cyclodextrin-mediated cholesterol depletion/enrichment of cultured cells**

Acute cholesterol depletion was carried out prior to fluorescent labelling by treating CHO-K1/A5 cells with 10-15 mM CDx or CDx-cholesterol complexes in Medium 1 (“M1”: 140 mM NaCl, 1 mM CaCl_2_, 1 mM MgCl_2_ and 5 mM KCl in 20 mM HEPES buffer, pH 7.4) essentially as in refs.[^2^](#_ENREF_2)^,^ [^3^](#_ENREF_3). Samples were taken at 20 min from culture dishes incubated at 37°C in the presence or absence of the cholesterol-modifying chemical.

**Fluorescence nanoscopy setup**

The optical nanoscope used, constructed in our laboratory, operates in the stochastic optical reconstruction microscopy (STORM)[^4^](#_ENREF_4) /ground state depletion microscopy followed by individual molecule return (GSDIM)[^5^](#_ENREF_5) modes. A 532 nm pumped solid state DPSS 300 mW laser (HB-Laserkomponenten GmbH, Schwäbisch Gmünd, Germany) was used as the excitation source, and delivered through a 0.65 FCP multiwavelength, polarization-maintaining fibre from Point Source, U.K. The output beam was passed through a dichroic filter (LLF532/10x Brightline, Semrock, Rochester, NY). The laser power was set to ~1.1 kW cm^-2^. Uniform epi-illumination of a field of view of ~10-30 μm in diameter was achieved by under-illuminating the back aperture of a plan-apochromatic TIRF 100x, 1.49 N.A. oil immersion objective (Nikon, Japan) mounted on a piezo objective Z-axis scanner (P-725 PIFOC, Physik Instrumente, Karlsruhe, Germany). A quarter-wave plate (375/550 nm, B. Halle Nachfolger GmbH, Berlin, Germany) was inserted into the illumination path to ensure nearly circular polarization of the laser beam. The fluorescence emitted by the sample was collected by the same objective lens and separated from the exciting laser light by a dichroic filter (Z580dcxr, AHF Analysentechnik, Tübingen, Germany). The 100x objective position was controlled along the Z-axis through a P-721.11 PIFOC nano-positioner and an E-662 objective controller from Physik Instrumente, Karlsruhe, Germany. Residual excitation laser light was removed by a notch filter (NF01-532U-25, AHF Analysentechnik, Tübingen, Germany) and the detection range was limited to the label emission spectrum by a single-band bandpass filter (for AlexaFluor^555^, we used a FF01-586/20 filter, Semrock, Rochester, NY). For Texas Red, the Nikon filter cube AT-TRITC LP filter cube was employed. The emission beam was magnified by 1.5x and imaged onto the back-illuminated electron multiplying CCD camera (iXon-Plus DU-860, Andor Technology, Belfast, Northern Ireland) driven at a gain of ~250, with a final pixel size of 106 nm, 3.6 photoelectrons per A/D count.

**Cell-surface fluorescence staining of nAChRs**

CHO-K1/A5 cells grown on 18 mm diameter No. 1.5 glass coverslips (WRL) in Ham’s F12 medium at 37°C were washed thrice with M1 medium, incubated in M1 medium for 45 min-1 h at 4°C with Alexa^555^-BTX at a final concentration of 1 μM and finally washed thrice with cold M1. Coverslips with the adhered cells were subsequently mounted in open holder chambers built at the Max-Planck-Institute for Biophysical Chemistry in Göttingen, Germany.

**Single-molecule superresolution imaging of live cells**

STORM imaging buffer[^6^](#_ENREF_6)^,^ [^7^](#_ENREF_7) consisting of 1% v/v glucose (500 mg/mL stock solution), 1% v/v glucose oxidase (5000 U/mL stock), 1% v/v catalase (40,000 U/mL stock) and 0.5% v/v 2-mercaptoethanol was filtered, degassed and UV irradiated under a transgel illuminator for 20 min prior to use to diminish background fluorescence. Coverslips with the cells stained with fluorescent BTX as described above were mounted in the custom-designed chambers for imaging at room temperature. The M1 physiological saline was replaced by the STORM imaging buffer just immediately before imaging proper. Cell viability is reported to be maintained for at least 20 min in this type of buffer[^8^](#_ENREF_8). Streams of single frames were acquired within the time window of <8 min as described below. All other imaging steps were carried out as described for fixed specimens. Cells were inspected after image acquisition to ensure preservation of cell morphology.

**Single-molecule superresolution imaging of fixed specimens**

CHO-K1/A5 cells were stained with mAb35 and subsequently with Alexa^555^- or Texas Red-labelled goat anti-mouse secondary antibody for 1 additional h at 4°C, and fixed with 2% paraformaldehyde containing 2% sucrose for 20 min at room temperature, washed thrice with M1 supplemented with 0.1M glycine containing 10% bovine foetal serum, covered with 20 μL of a 1% aqueous solution of 25,000 MW PVA in Millipore-filtered distilled water, spun in a table top centrifuge rotor shaft to form a thin layer of PVA [^9^](#_ENREF_9), and finally mounted onto the holder chambers. Alternatively, a drop of Prolong Gold was added to the coverslip and the latter was allowed to settle overnight onto a glass slide. Cells were initially inspected with LED excitation under low intensity illumination conditions and appropriate areas were selected for STORM imaging. Illumination was then switched to the high-intensity laser source, and the CCD camera was set to acquire a stream of images at maximum frame rate. Between 7,000 and 10,000 frames were acquired at rates of ~10 ms/frame from the ventral, coverslip-adhered surface of 8-15 cells for each experimental condition with an Andor iXon Plus DU-860 EM-CCD camera with a 1.0 or a 1.5x projection lens, yielding a pixel size of 160 nm or 106 nm in the image plane, respectively. Streaming movies were acquired using the software SlideBook (Intelligent Imaging Innovations, Boulder, CO) and exported as 16-bit TIF or Matlab files for subsequent off-line analysis.

**Superresolution data analysis**

**i) Determination of sub-diffraction molecular coordinates**

The off-line localization of the x,y coordinates of the nAChR spots was carried out using the image analysis package ThunderSTORM (<https://code.google.com/p/thunder-storm/>) [^10^](#_ENREF_10) run as a plugin in ImageJ (https://imagej.nih.gov/ij/). ThunderSTORM is particularly suitable for separating multiple overlapping PSFs (typical emitter density was 3.78 ± 0.01; see Suppl. Fig. 1c). To account for the discrete nature of pixels in digital cameras, an integrated form of a symmetric 2D Gaussian function was fitted to the spots using Levenberg–Marquardt least-squares minimization routines. Localizations that were too close together to be independent were discarded. The ThunderSTORM multi-emitter fitting analysis was enabled, and the limiting intensity range was set at 500-2,000 photons. Other ThunderSTORM filters were enabled to remove uncertainty-based duplicates (e.g. multiple emitters, duplicate localizations, as described in ref. [^11^](#_ENREF_11)). Lateral drift was estimated experimentally using fiducial 100 nm coverslip-adhered fluorescent beads and corrected via the appropriate filter in ThunderSTORM. Localization precision was calculated automatically via ThunderSTORM using a modified version of the formula in ref. [^12^](#_ENREF_12) which considers the EM gain of the EM-CCD camera (Quan et al. 2010), through the following expression:

$\left\langle\left( \Delta x \right)^{2} \right\rangle=\frac{2\sigma^{2}+{a^{2}}/{12}}{N}+\frac{8\pi\sigma^{4}b^{2}}{a^{2}N^{2}}$ (1)

Where $\sigma$ is the standard deviation of the fitted point spread function (PSF), $a$ is the pixel size in nm, $N$ is the intensity expressed in number of photons and $b$ is the background signal level in photons. The average localization precision was 37.58 ± 0.02 nm (see Suppl. Fig. 1a).

**ii) Single-particle tracking (SPT)**

The fluorescent particles were detected by a generalized likelihood ratio test algorithm specifically designed to detect point spread function-shaped (i.e. Gaussian-like) spots using ThunderSTORM [^10^](#_ENREF_10) and exported in a format suitable for tracking analysis using an ad-hoc Matlab routine written in our laboratory. Detected (validated) particles were further analyzed for their trajectories with the software package Localizer (https://bitbucket.org/pdedecker/localizer)^[13](#_ENREF_13)^ implemented in Igor Pro (Wavemetrics Inc. https://www.wavemetrics.com). Two critical parameters were set in Localizer: the maximal number of frames (3 = 30 ms) that a given molecule was allowed to blink (“Max blinking”), and the maximum distance (“Max jump distance”) at which two points could lie and be attributed to the same trajectory (3 pixels). The optimal “Max blinking” ($t_{off}$) was determined following the method of Annibale and coworkers [^14^](#_ENREF_14), which implies that the number of photoblinking fluorescent molecules $N$ in the sample can be estimated from the number of counts (localizations) at different dark times $t_{d}$, $N(t_{d})$, by fitting to the semi-empirical equation:

$N\left( t_{d} \right)=N\left( 1+n_{blink}e^{\left( \frac{1-t_{d}}{t_{off}} \right)} \right)$ (2)

in the regime of low dark time $t_{d}$ values [^14^](#_ENREF_14). The maximum distance for the merging filter in our experiments was determined from the camera pixel size and the distance-filter criterion described by others[^15^](#_ENREF_15). Briefly, localized molecules that reappeared in consecutive frames were considered as corresponding to the same molecule if the frame-to-frame displacement (tracking radius) was within 106 nm, thus allowing the monitoring of molecules with diffusion coefficients of up to 1.33 µm^2^ s^-1^, i.e. conservatively higher than the upper bound for nAChR nanocluster diffusion estimated from TIRF-SPT experiments in our laboratory[^3^](#_ENREF_3)

. Following the above argument, the “Max jump distance” was deduced by considering the maximum distance that a nAChR is allowed to “jump” within the merge time window established above, i.e. the maximum distance that, on average, a single-molecule can travel in 3 frames.

**iii) Mean-square displacement (MSD) analysis**

Typically, a diffusion process is characterized by the time-averaged mean-square displacement (tMSD), which for a 2-dimensional space like a membrane bilayer can be written as:

$\mathrm{MSD}= <{\Delta r}^{2}\left( t \right)> = \int_{-\infty}^{\infty} r^{2}(t)P\left( r,t \right) d^{2}r=4Dt$ (3)

Where *D* is the diffusion constant. This assumes a purely viscous and homogeneous fluid, such that $P(r,t)$ is the probability distribution function (PDF, also termed propagator) of the diffusion process, i.e., the probability of finding the particle at a (radial) distance *r* away from the origin at time *t* after release of the particle at *r* = 0 at time *t* = 0.

Complex media may lead to sublinearity of the MSD as a function of time:

$\mathrm{MSD}= <{\Delta r}^{2}\left( t \right)> = K_{\beta}t^{\beta}$ (4)

In equation 3, anomalous diffusion is taken into account by introduction of the exponent $\beta$ [^16^](#_ENREF_16)^,^ [^17^](#_ENREF_17), where $\left( MSD \right) \sim t^{\beta}$. Whereas for $\beta=1$ simple thermally-driven (“random walk”) Brownian diffusion results, two forms of anomalous diffusion result from other values of $\beta$: subdiffusion for $0<\beta<1$ (e.g. in molecular crowding), and superdiffusion for $\beta>1$. Thus, in the case of anomalous diffusion Eq. (4) above can be written in short-form as: 4*Dt*^β^.

The time-averaged mean-square displacement (tMSD) was calculated for each trajectory $j$ in the form:

$\mathrm{tMSD} \left( t_{lag}=m\Delta t \right)= {<{\Delta r}^{2}\left( t_{lag}=m\Delta t \right)>}_{T} =\frac{1}{M}\sum_{i=1}^{T/{\Delta t}} {R_{j}}^{2}\left( t_{i}+m\Delta t \right)$ (5)

${R_{j}}^{2}\left( t_{i}+m\Delta t \right)={[x_{j}\left( t_{i}+m\Delta t \right)-x_{j}(t_{i})]}^{2}+{[y_{j}\left( t_{i}+m\Delta t \right)-y_{j}(t_{i})]}^{2}$ (6)

where $(x_{j},y_{j})$ is the position sampled at $M$ discrete times $t_{i}=i\Delta t$ with displacements different from NaN, $\Delta t$ is the acquisition time (in our case, $\Delta t=10 ms$), T is the total averaging time and $i$ is the frame number.

The ensemble-averaged mean-square displacement (eMSD) was calculated over a time interval $m\Delta t$,

$\mathrm{eMSD} \left( t_{lag}=m\Delta t \right)={<{\Delta r}^{2}\left( t_{lag}=m\Delta t \right)>}_{ens}=\frac{1}{N}\sum_{j=1}^{N} {R_{j}}^{2}\left( t_{i}+m\Delta t \right)$ (7)

where $N$ is the total number of available single-molecule trajectories (not NaN) at time $t_{i}$, where $t_{i}$ is the starting time relative to the first point in the trajectory. Here we follow the nomenclature employed by Krapf and coworkers and the calculation of the tMSD and eMSD was done following their procedures[^18^](#_ENREF_18). Following the criteria of ref.[^19^](#_ENREF_19) the power (anomalous) exponent $\beta$ was obtained by linear fitting the initial 50 points of the log-log transformed tMSD and the eMSD, respectively. The generalized diffusion coefficient, $K_{\beta}$, was obtained from the linear fit to the first 50 time points of the individual eMSDs in log-log scale, evaluated at $t=1$ (see Eq. 4), also following the criteria of ref.[^19^](#_ENREF_19).

**iv) Exclusion of immobile particles in the analysis of nAChR trajectories**

Stationary (immobile) molecules were excluded from the analysis of single-molecule trajectories following a series of recently introduced criteria[^19^](#_ENREF_19). The procedure sets a threshold value on the ratio of the radius of gyration $R_{g}$and the mean step size $\left| \Delta r \right|$of the particles´displacement. In the case of ideal immobile particles this ratio is constant, whereas for mobile particles the ratio increases. The normalized ratio:

($\sqrt{\pi/2} {(R_{g}}/\left\langle\left| \Delta r \right| \right\rangle)$ (8)

was obtained from experiments with paraformaldehyde-fixed cells, and the ratio was subsequently employed to obtain the threshold value applied to live cells experiments. [Golan and Sherman (2017](#_ENREF_24)) discuss the advantages of this method over the use of the diffusion coefficient or $R_{g}$alone for excluding immobile particles; the two latter procedures would falsely classify immobile particles as mobile. Threshold values with >95% confidence were obtained by pooling data from different cells in independent sets of experiments.

**v) Classification of mobile particles according to their diffusivity and ergodicity analysis**

To categorize mobile trajectories into subpopulations according to their diffusivity, we first plotted the tMSDs in a log-log scale. Linear fitting of the curves rendered the power (anomalous) exponent β and the goodness of the fit. Those having a goodness of fit better than 0.9 were selected. The anomalous exponent β was then used to classify trajectories into 5 arbitrary subpopulations: subdiffusive I (β < 0.5), subdiffusive II (0.5 ≤ β< 0.7), subdiffusive III (0.7 ≤ β< 0.9), Brownian (0.9 ≤ β < 1.1), and superdiffusive (β ≥ 1.1).

In statistical mechanics, the ergodic theory predicts that for large systems of interacting particles at equilibrium, the time average along a single trajectory equals the ensemble average (i.e. for sufficiently long measurement times, the time average provides the same information as the ensemble average). Thus, the observed diffusion coefficient obtained from an individual trajectory is identical to the diffusion constant found from an ensemble of particles under identical physical conditions. This equivalence between the ensemble diffusion and the behaviour of an individual representative particle indicates that the process is ergodic (see recent review and references therein in ref.[^20^](#_ENREF_20)). When inequality holds at long measuring times it is assumed that the process violates the Botzmann-Khinchin ergodic hypothesis[^21^](#_ENREF_21)^,^[^22^](#_ENREF_22). To determine whether the nAChR motion at the cell surface was ergodic or not, the time- and ensemble-averaged mean-square displacements (tMSD and eMSD, respectively) were calculated for each experimental condition and the anomalous exponent β was obtained as described above.

**vi) Escape time distributions**

The escape (waiting) time is the interval during which a trajectory remains within a given radius *R_TH_*. The duration of the events in which the molecules’ trajectories remained within circular areas of increasing radii *R_TH_* was calculated as in ref.[^18^](#_ENREF_18). Briefly, for each trajectory $j$:

a) We generated the displacement squares

${R_{t_{lag}=\Delta t,i,j}}^{2}$ (9)

for each time $t_{i}=i\Delta t$, where $\Delta t$ corresponds to the inter-frame time (in our case, $\Delta t=10 \mathrm{ms}$).

b) Next, we looked for the first displacement for which

${R_{t_{lag}=\Delta t,i,j}}^{2}<{R_{TH}}^{2}$ (10)

We called this time $t_{k}=k\Delta t$, i.e. the time corresponding to the frame $k$ at lag time $t_{lag}=\Delta t$.

c) Next, we observed the displacement squares at times $t_{k}$ for increasing lag times $t_{lag}=2\Delta t,3\Delta t,\ldots$ until either the relation ${R_{t_{lag},k,j}}^{2}>{R_{TH}}^{2}$was satisfied or the trajectory ended. This is true for a certain escape time

$t_{lag}=t_{F}=F\Delta t$ (11)

d) When the escape time was obtained, we continued from $t_{k}+t_{F}+\Delta t=t_{k+F+1}$ looking for the next displacement that satisfied the relationship:

${R_{\Delta t,i,j}}^{2}<{R_{TH}}^{2}$ (12)

for $(i\geq k+F+1$)

e) If the step was found, steps (b-c) were repeated until the end of the trajectory.

Finally, we constructed the cumulative distribution function for all the resulting escape times.

**vii) Turning angle (directional change) analysis**

The turning angle distribution as a function of the time lags has been employed to search for correlations in the particles´ displacements. We applied this analysis to follow the directional changes in individual nAChR trajectories using the relative angles distended by the molecules along their walk, a parameter which can help distinguish among different types of anomalous subdiffusive mechanisms, among them, the so-called fractional Brownian motion (fBM) and obstructed diffusion models[^23^](#_ENREF_23).

**viii) Single-molecule trajectory recurrence analysis**

The presence of confinement periods within individual trajectories was identified using recently developed algorithms[^24^](#_ENREF_24), based on the evaluation of the total number of visits (recurrence) performed by the moving particle to a given site. Confinement within a nanoscale domain is associated with multiple visits to the same site, multiple times, in a short period, whereas unconfined motion is related to exploration of wider regions, less compact walks, and less visits to the sites previously walked. The analyses characterize subpopulations of trajectories based on the times spent in each state and the areas distended in confinement.

**ix) Identification of dynamic nAChR nanoclusters**

We applied the centroid-linkage hierarchical clustering and the density-based spatial clustering with noise (DBSCAN) analyses embedded in the open source qSR software developed by Cissé and coworkers^25^ ([www.github.com/cisselab/qSR](http://www.github.com/cisselab/qSR)) to identify in real time the formation and breakage of clusters of nAChR molecules within the confined zones. The length scale was set at 100 nm, and the minimal number of particles was 20 in the DBSCAN analysis. Dark-time tolerance was set at 1 s. All localizations were included in this analysis, and only for this purpose.

**Statistical analyses**

These were done using one-way analysis of variance employing ANOVA or Kruskal-Wallis, where appropriate. Multiple comparisons tests were performed using Tukey’s or Dunn’s approaches implemented in the Prism GraphPad software. Mean ± 95% confidence intervals are shown unless otherwise stated. The one-sample Kolmogorov-Smirnov test was applied to assess whether the data were normally distributed or not. To compare two distributions, we used the Kolmogorov-Smirnov (KS) test for two samples.

**Results**

**Localization precision and merge time**

**
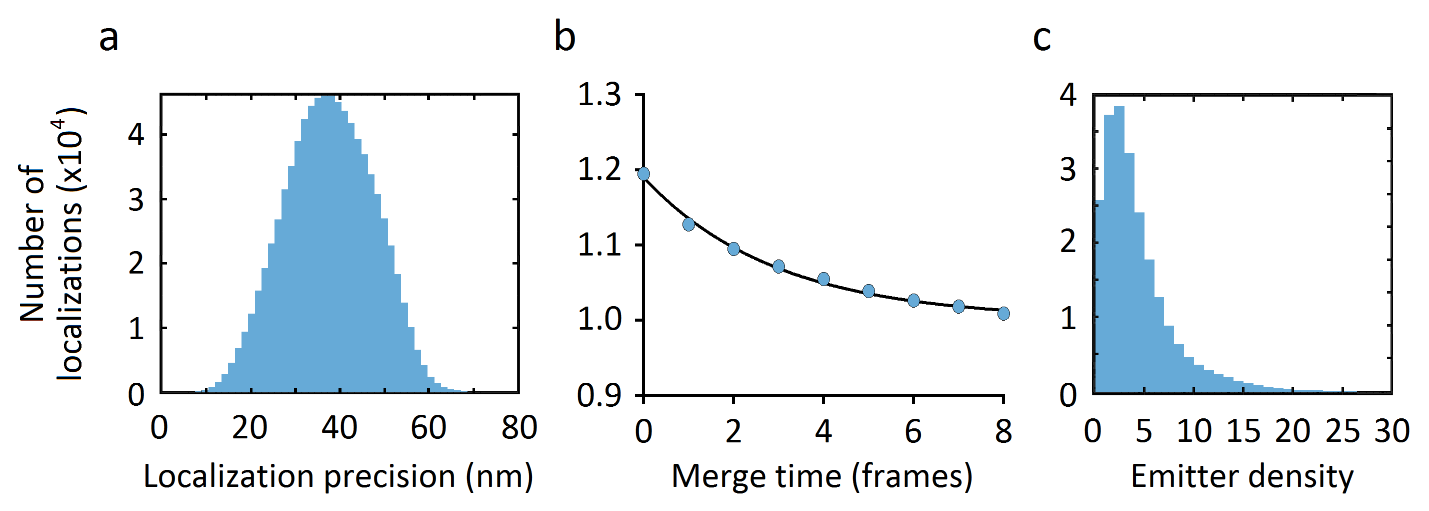
**The localization precision and merge time are two essential parameters in STORM and in single-molecule localization microscopy in general. Suppl. Figure 1 shows the histograms for determining the localization precision as applied to BTX-labelled nAChR molecules.

**Supplementary Figure 1. Localization precision and merge time**. a) Histogram of the localization precision using the method of Quan and coworkers[^26^](#_ENREF_26) (see eq. (1) ) as applied to two CHO-K1/A5 cells labelled with BTX. The average localization precision was 37.58 ± 0.02 nm. b) Determination of the optimal merge time following the method of Annibale and coworkers [^14^](#_ENREF_14). The plot shows the total number of localizations against the merge time in a representative image of BTX-labelled nAChRs and the corresponding fit to the semi-empirical Eq. (2). In the example shown, the optimal merge time was found to be three frames (30 ms). c) Normalized histogram of the number of localized molecules per frame, i.e. the emitter density. The typical emitter density was 3.78 ± 0.01.

**Classification of nAChR tracks into mobile and immobile trajectories.**

Suppl. Figure 2 shows the ratio of the radius of gyration $R_{g}$and the mean step size (a), examples of mobile and immobile particles (b), and the experimentally determined percentage of immobile particles upon application of the Golan and Sherman’s criteria[^19^](#_ENREF_19). The combination of the radius of gyration and the mean displacements of nAChR validated localizations in paraformaldehyde-fixed cells (Suppl. Figure 2a) led us to set a threshold value of 2.1 which was used as the cutoff for exclusion of immobile particles. This enabled us to calculate the relative proportion of mobile/immobile particles in live speciments (Suppl. Figure 2), and exclude immobile molecules from further analysis. The CHO-K1/A5 mammalian clonal cell line lacks receptor-immobilizing molecules like rapsyn or clustering non-receptor scaffolding proteins like agrin and MusK. Immobilization must therefore respond to other scaffolding molecules or the self-aggregation of the nAChR protein in higher oligomeric species[^27^](#_ENREF_27), or a combination thereof.


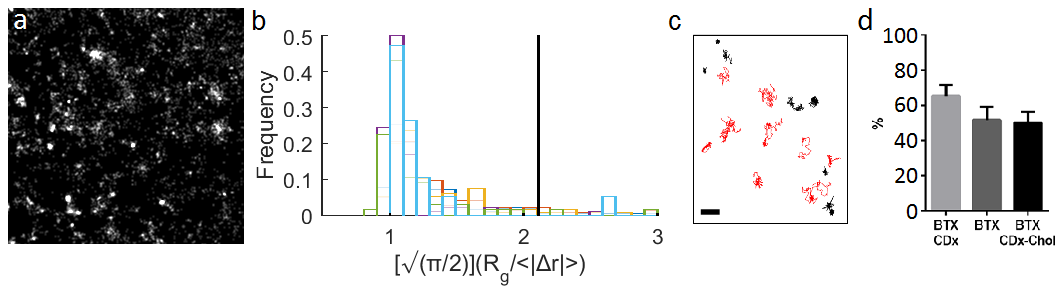


**Supplementary Figure 2. Criteria for classification of nAChR trajectories into mobile and immobile.** a) A 5 x 5 μm Gaussian-smoothened projection image of a fixed CHO-K1/A5 cell stained with mAb35 followed by Texas Red-labelled goat secondary antibody, displaying the single-molecule localizations. ~~a~~b) Distribution of the ratio of the radius of gyration and the mean step size for two independent samples of nAChRs in paraformaldehyde-fixed cells (n=6). Threshold values of 1.5-2.1 (95% confidence) were obtained[^19^](#_ENREF_19) and the value of 2.1 was chosen (black line). c) Mobile (red) and immobile (black) nAChR trajectories under control conditions resulting from application of the criteria of ref.[^19^](#_ENREF_19) for separating particle walks into these two categories. Scale bar: 500 nm. d) Comparison of the percentages of immobile nAChR trajectories upon application of the threshold resulting from Suppl. Figure 2a. Bars indicate mean ± S.E.M.

**Modification of cholesterol levels of CHO-K1/A5 cells**

Methyl-β-cyclodextrin (CDx) is a widely used tool to acutely modify the cholesterol levels at the plasma membrane. The kinetics and thermodynamics of cholesterol efflux from /incorporation into the membrane are relatively well understood: once CDx establishes contact with the plasmalemma, cholesterol migrates from the membrane to the CDx core [^28^](#_ENREF_28) [^29^](#_ENREF_29). We have used CDx alone, or in complex with cholesterol, to deplete (CDx) or replenish (CDx-Chol), respectively, the cell-surface sterol content and assess cholesterol effects on receptor transport mechanisms[^30^](#_ENREF_30)^,^ [^31^](#_ENREF_31), nAChR diffusion[^3^](#_ENREF_3)^,^ [^32^](#_ENREF_32)^,^ [^33^](#_ENREF_33) and cell-surface organization[^34^](#_ENREF_34). Here we applied CDx or CDx-Chol and analysed the effects on the nAChR at the ensemble level or at the level of trajectories divided into subpopulations according to diffusivity. Suppl. Table 1 below lists the percentage of trajectories found in each subpopulation under control and cholesterol modifying conditions.

**Supplementary Table 1. Percentage of trajectories in subpopulations separated according to their power (anomalous) exponent β* under control and cholesterol-modifying conditions****

| **Treatment** | **Subdiffusive I** | **Subdiffusive II** | **Subdiffusive III** | **Brownian** | **Superdiffusive** |
| --- | --- | --- | --- | --- | --- |
| CDx | 10.75 ± 7.64 | 27.7 ± 8.71 | 31.04 ± 10.15 | 12.80 ± 9.74 | 3.24 ± 3.51 |
| Control | 8.63 ± 5.90 | 31.91 ± 11.65 | 30.08 ± 15.99 | 22.39 ± 13.76 | 3.08 ± 4.03 |
| CDx-Chol | 5.67 ± 8.41 | 25.79 ± 18.66 | 33.39 ± 9.84 | 20.34 ± 10.35 | 10.07 ± 8.27 |

*Groups are ordered from less diffusive (left) to more diffusive (right). Diffusivity-based classification of trajectories (see main text) rendered the following groups: subdiffusive I (β < 0.5), subdiffusive II (0.5 ≤ β< 0.7), subdiffusive III (0.7 ≤ β< 0.9), Brownian motion (0.9 ≤ β < 1.1), and superdiffusive (β ≥ 1.1). Values are the mean ± S.D. Statistically significant differences between BTX control and BTX CDx-Chol superdiffusive (p=0.042). **Trajectories not satisfying a goodness of fit of 0.9 are not included in this Table. They represented a variable proportion of the total, amounting to ~4-14%.

**Time-averaged mean-square displacement (tMSD) of nAChR trajectories**

The mean-square displacements (MSDs) of the mobile trajectories were analysed in the total unsorted population of nAChRs (Figure 2 in main text). Their ergodic and non-ergodic behaviour can be appreciated from inspection of the values of the anomalous exponent β (Table 1 in main text). The plots in Supplementary Figure 3 below illustrate the MSD curves for the subpopulations separated according to diffusivity.


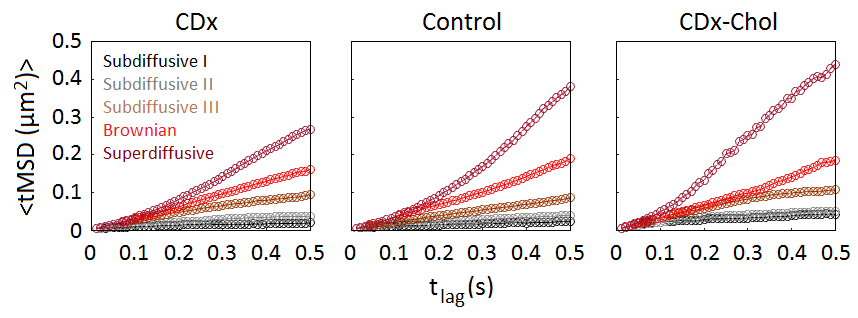


**Supplementary Figure 3.** **Average time-averaged mean-square displacement (tMSD) of nAChR trajectories separated into subpopulations according to their power (anomalous) exponent β.** Log-log plots of the average tMSD having a goodness of fit better than 0.9 (see Material and Methods). Each colour corresponds to a different diffusivity-based subpopulation ordered according to its power exponent.


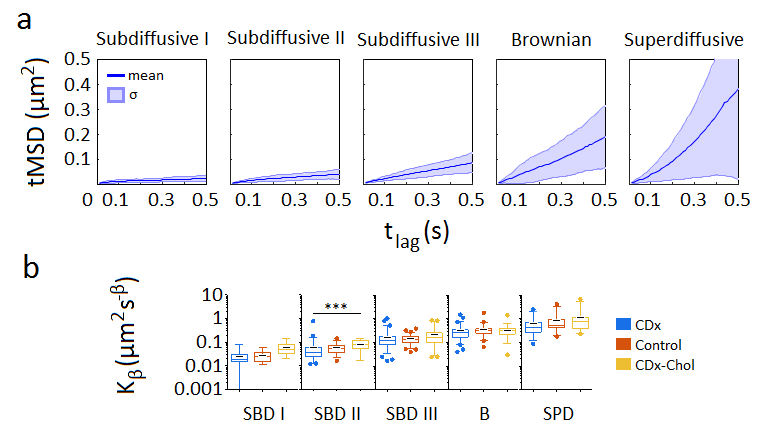


**Supplementary Figure 4.** a) Average tMSDs of nAChR trajectories separated according to diffusivity.  b) Generalized diffusion coefficients, $K_{\beta}$, of the subpopulations of trajectories classified according to their anomalous exponent β (plots are ordered according to increasing β ranges as shown above in (a)). Whiskers in box plots correspond to 95% confidence intervals. The limits indicate 75% confidence intervals; the black + symbols indicate the mean and the horizontal lines the median in each case. The dots outside the confidence intervals are outliers. Statistics: (***), p<0.001.

**Turning angle analysis**.

Turning angle analysis was recently employed to study the correlation of experimentally determined single-molecule steps in voltage-gated potassium channels Kv1.4 and Kv2.1 [^35^](#_ENREF_35) in comparison to numerical simulations of fractional Brownian motion (fBM) and obstructed diffusion (OD) models. A schematic diagram of this type of analysis is shown in Suppl. Figure 5. Experimental results corresponding to the cholesterol-modified nAChR subpopulations separated according to diffusivity are shown in Suppl. Figure 6.

**
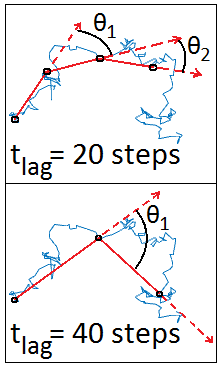
**

**Supplementary Figure 5.** Schematic diagram depicting the procedure in turning angle analysis [^23^](#_ENREF_23). At the starting position the observer follows the trajectory (blue trace) with a given number of steps (20 or 40 in the example shown) defining the time-lag (t_lag_), and the angles (θ_1_, θ_2_….) distended by successive steps.

**
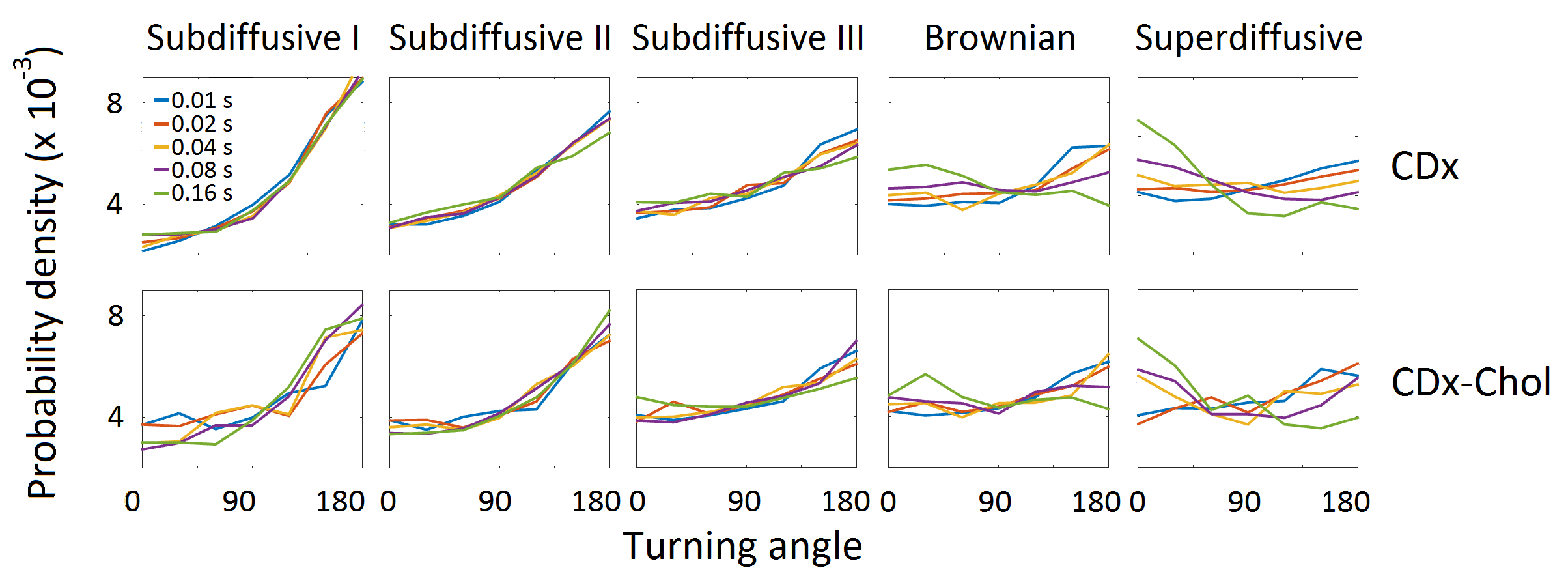
**

**Supplementary Figure 6. Turning angle probability densities for the diffusivity-classified trajectories under cholesterol depletion (CDx) and cholesterol-enrichment (CDx-Chol) conditions.** Probability densities for the colour-coded t_lag_ of increasing durations (10 to 160 ms). The probability density is normalized such that the integral of the curve is equal to unity.

**Transient confinement nanodomains within individual trajectories and differences between subdiffusive states**

The results of the recurrence analysis[^36^](#_ENREF_36) as applied to the individual trajectories in the diffusivity-based subpopulations under control conditions are shown in the main text (Figure 5). This analysis disclosed microscopic heterogeneity in all trajectories and differences in diffusion characteristics among subpopulations. Suppl. Figure 7a shows the differences in the areas transiently occupied by the trajectories in their confined portion. The areas covered by the confined section of the trajectories show statistically significant differences between all the subdiffusive subpopulations (which are similar to each other) and the Brownian trajectories (p < 0.0001). The cumulative distribution times (Suppl. Figure 7b) show differences between different subpopulations, which are attenuated for the faster diffusional regimes. In the subdiffusive I subpopulation, a marked shortening of the characteristic decay time is observed upon cholesterol modification (Supplementary Table 2). This is in marked contrast with the Brownian subpopulation, in which case cholesterol modification appears to lengthen the residence time in the confined state (Supplementary Table 2).

**
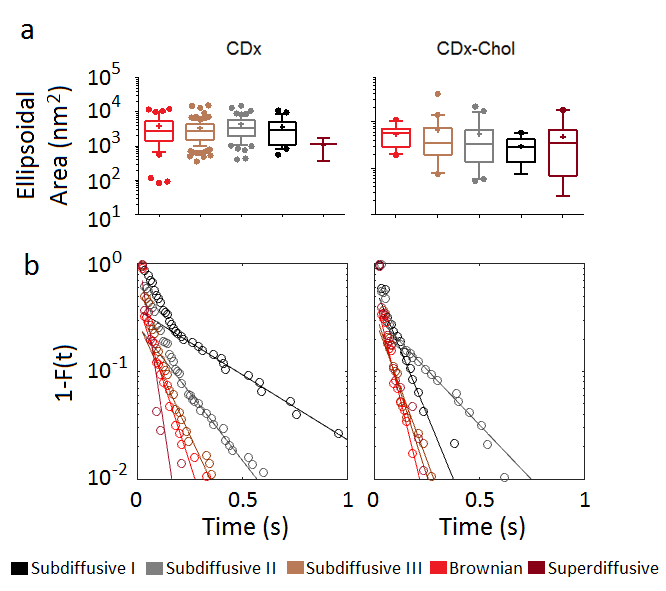
**

**Supplementary Figure 7. Confinement areas and complementary cumulative distribution function of the residence times in the confined state under cholesterol modification conditions for each diffusivity-based group.** a) Mean ellipsoidal area of the trajectories´ portion in the confined state. b) The sojourn times have an exponential distribution tail with a characteristic decay time, which turns out to be inversely proportional with the subpopulation diffusivity (see Suppl. Table 2 below).

**Supplementary Table 2. Decay times of the confined portions corresponding to the individual trajectories in subpopulations separated according to their power (anomalous) exponent β and in the entire population*.**

| ***Subpopulation*** | **Subdiffusive I** | | **Subdiffusive II** | | **Subdiffusive III** | **Brownian** | **Superdiffusive** |
| --- | --- | --- | --- | --- | --- | --- | --- |
| **CDx** | | | | | | | |
|  | |  | |  |  |  |  |
| **Tau** (s) | | 0.361 ± 0.033 | | 0.174 ± 0.009 | 0.103 ± 0.009 | 0.073 ± 0.007 | 0.033 ± 0.013 |
| **Control** | | | | | | | |
|  | |  | |  |  |  |  |
| **Tau** (s) | | 0.310 ± 0.022 | | 0.129 ± 0.012 | 0.116 ± 0.008 | 0.041 ± 0.003 | 0.045 ± 0.014 |
| **CDx-Chol** | | | | | | | |
|  | |  | |  |  |  |  |
| **Tau** (s) | | 0.097 ± 0.044 | | 0.213 ± 0.012 | 0.060 ± 0.012 | 0.044 ± 0.008 | 0.055 ± 0.008 |

| ***Total Population*** | **CDx** | **Control** | **CDx-Chol** |
| --- | --- | --- | --- |
|  |  |  |  |
| **Tau** (s) | 0.257 ± 0.007 | 0.171 ± 0.009 | 0.135 ± 0.005 |

The question arose as to whether the different diffusional regimes are given by the concentration of obstacles -as suggested by the progressive decrese in the slope of the turning angles (Figure 4b and Suppl. Figure 6) or, in contrast, whether they result from the averaging of the lifetimes of confined and free regions of the individul trajectories. Suppl. Figure 8 below shows that the slope of the turning angle in the confined portion of the individual trajectories is essentially the same, confirming the idea that the concentration of obstacles is the same for all the confined regions independently of the ensemble diffusional regime; it is the time spent in one state or the other which determines the diffusional modality, as supported by the clear correlation between the diffusivity-based subpopulations with the percentage of single-molecule confinement and the subsequent disappearance of subdiffusive motifs in the free portions of the trajectories, as shown in Supplementary Figure 9 below.

**
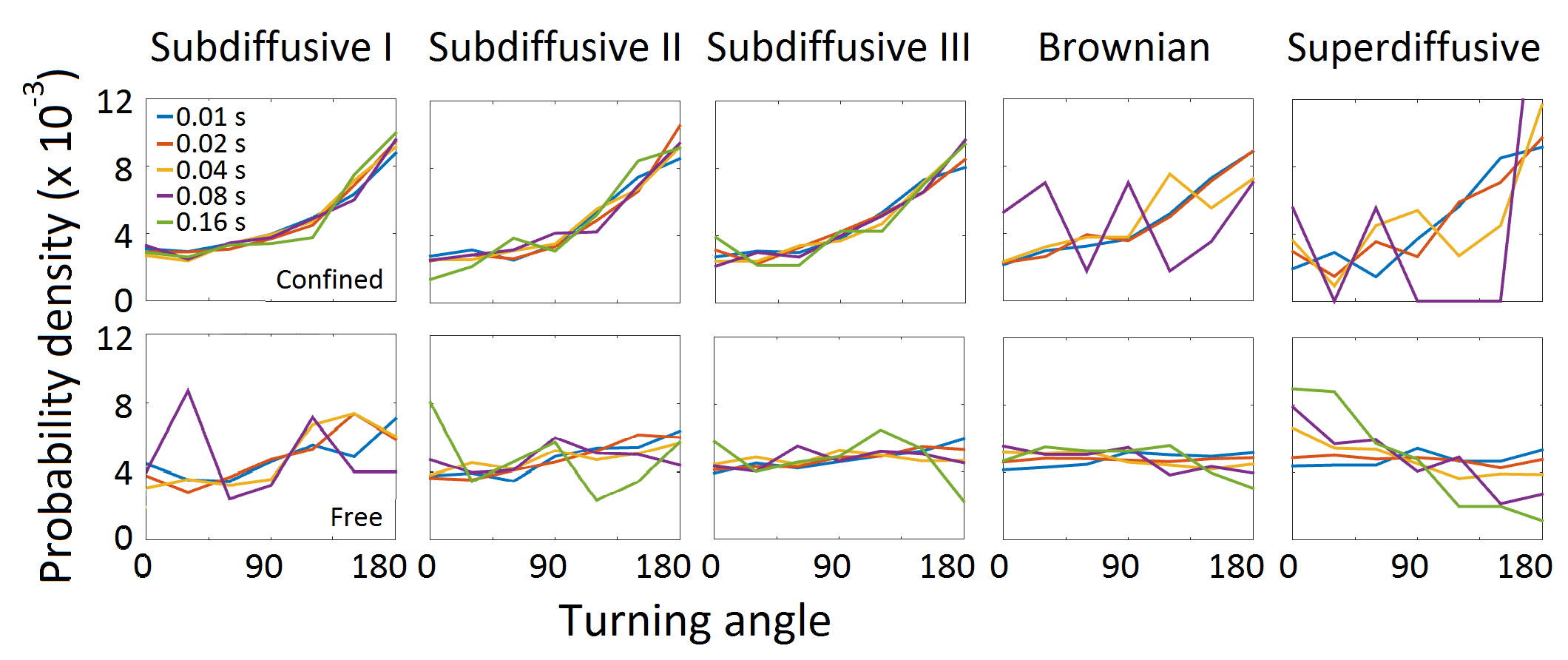
**

**Supplementary Figure 8. Turning angle probability densities of the transiently confined and unconfined portions of the individual trajectories under control conditions.** Probability densities for the colour-coded t_lag_ of increasing durations (10 to 160 ms). The probability density is normalized such that the integral of the curve is equal to unity.


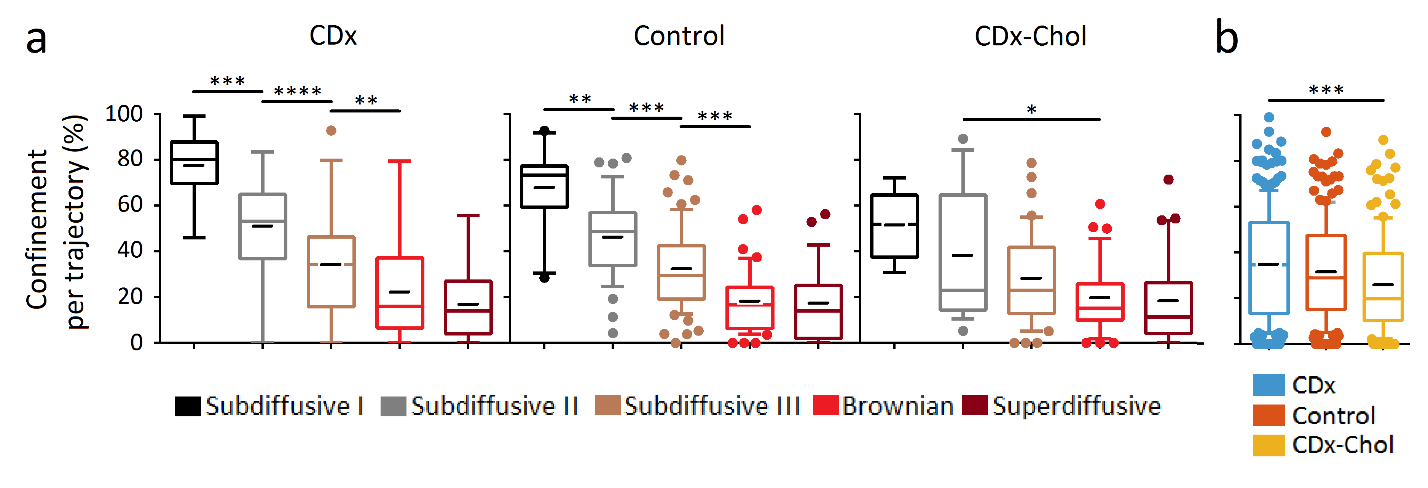


**Supplementary Figure 9**. **Percentage of confinement events in diffusivity-classified single-molecules and anomalous exponent β distributions in free and confined portions of the total population of trajectories.** a) Confinement per single-molecule trajectory in diffusivity-based subpopulations and b) for the entire population. p-values: (*), p<0.05; (**), p<0.01; (***), p<0.001 and (****), p<0.0001.

**References**

1. Roccamo, A.M. *et al.* Cells defective in sphingolipids biosynthesis express low amounts of muscle nicotinic acetylcholine receptor. *The European journal of neuroscience* **11**, 1615-1623 (1999).

2. Borroni, V. *et al.* Cholesterol depletion activates rapid internalization of submicron-sized acetylcholine receptor domains at the cell membrane. *Mol.Membr.Biol.* **24**, 1-15 (2007).

3. Almarza, G., Sanchez, F. & Barrantes, F.J. Transient cholesterol effects on nicotinic acetylcholine receptor cell-surface mobility. *PloS one* **9**, e100346 (2014).

4. Rust, M.J., Bates, M. & Zhuang, X. Sub-diffraction-limit imaging by stochastic optical reconstruction microscopy (STORM). *Nat.Methods* **3**, 793-795 (2006).

5. Folling, J. *et al.* Fluorescence nanoscopy by ground-state depletion and single-molecule return. *Nat.Methods* **5**, 943-945 (2008).

6. Harada, Y., Sakurada, K., Aoki, T., Thomas, D.D. & Yanagida, T. Mechanochemical coupling in actomyosin energy transduction studied by in vitro movement assay. *J Mol Biol* **216**, 49-68 (1990).

7. Adachi, K. *et al.* Stepping rotation of F1-ATPase visualized through angle-resolved single-fluorophore imaging. *Proc Natl Acad Sci U S A* **97**, 7243-7247 (2000).

8. Jones, S.A., Shim, S.H., He, J. & Zhuang, X. Fast, three-dimensional super-resolution imaging of live cells. *Nat. Methods* **8**, 499-508 (2011).

9. Barrantes, F.J. Single-molecule localization superresolution microscopy of synaptic proteins., in *Springer Protocols*, Edn. 2016. (ed. A.K. Shukla) 1-42 (Springer Science+Business Media, 2016).

10. Ovesný, M., Krizek, P., Borkovec, J., Svindrych, Z. & Hagen, G.M. ThunderSTORM: a comprehensive ImageJ plug-in for PALM and STORM data analysis and super-resolution imaging. *Bioinformatics (Oxford, England)* **30**, 2389-2390 (2014).

11. Huang, F., Schwartz, S.L., Byars, J.M. & Lidke, K.A. Simultaneous multiple-emitter fitting for single molecule super-resolution imaging. *Biomedical optics express* **2**, 1377-1393 (2011).

12. Thompson, R.E., Larson, D.R. & Webb, W.W. Precise nanometer localization analysis for individual fluorescent probes. *Biophys.J* **82**, 2775-2783 (2002).

13. Dedecker, P., Duwé, S., Neely, R.K. & Zhang, J. Localizer: fast, accurate, open-source, and modular software package for superresolution microscopy. *Journal of biomedical optics* **17**, 126008-126008 (2012).

14. Annibale, P., Vanni, S., Scarselli, M., Rothlisberger, U. & Radenovic, A. Quantitative photo activated localization microscopy: unraveling the effects of photoblinking. *PloS one* **6**, e22678 (2011).

15. Lu, H.E., MacGillavry, H.D., Frost, N.A. & Blanpied, T.A. Multiple spatial and kinetic subpopulations of CaMKII in spines and dendrites as resolved by single-molecule tracking PALM. *The Journal of neuroscience : the official journal of the Society for Neuroscience* **34**, 7600-7610 (2014).

16. Tejedor, V. *et al.* Quantitative analysis of single particle trajectories: mean maximal excursion method. *Biophys.J* **98**, 1364-1372 (2010).

17. Manzo, C. *et al.* Weak ergodicity breaking of receptor motion in living cells stemming from random diffusivity. *Physical Review X* **5**, 011021 (2015).

18. Weigel, A.V., Simon, B., Tamkun, M.M. & Krapf, D. Ergodic and nonergodic processes coexist in the plasma membrane as observed by single-molecule tracking. *Proc Natl Acad Sci U S A* **108**, 6438-6443 (2011).

19. Golan, Y. & Sherman, E. Resolving mixed mechanisms of protein subdiffusion at the T cell plasma membrane. *Nature communications* **8**, 15851 (2017).

20. Metzler, R., Jeon, J.H. & Cherstvy, A.G. Non-Brownian diffusion in lipid membranes: Experiments and simulations. *Biochim Biophys Acta* **1858**, 2451-2467 (2016).

21. Burov, S., Metzler, R. & Barkai, E. Aging and nonergodicity beyond the Khinchin theorem. *Proc Natl Acad Sci U S A* **107**, 13228-13233 (2010).

22. Cherstvy, A.G. & Metzler, R. Anomalous diffusion in time-fluctuating non-stationary diffusivity landscapes. *Physical chemistry chemical physics : PCCP* **18**, 23840-23852 (2016).

23. Burov, S. *et al.* Distribution of directional change as a signature of complex dynamics. *Proc Natl Acad Sci U S A* **110**, 19689-19694 (2013).

24. Sikora, G. *et al.* Elucidating distinct ion channel populations on the surface of hippocampal neuros via single-particle tracking recurrence analysis. *arXiv:1708.02876* (2017).

25. Andrews, J.O. *et al.* qSR: A software for quantitative analysis of single molecule and super-resolution data. *bioRxiv* (2017).

26. Quan, T. *et al.* Ultra-fast, high-precision image analysis for localization-based super resolution microscopy. *Opt Express* **18**, 11867-11876 (2010).

27. Barrantes, F.J. Oligomeric forms of the membrane-bound acetylcholine receptor disclosed upon extraction of the Mr 43,000 nonreceptor peptide. *J.Cell Biol.* **92**, 60-68 (1982).

28. Kilsdonk, E.P.C. *et al.* Cellular cholesterol efflux mediated by cyclodextrins. *J.Biol.Chem.* **270**, 17250-17256 (1995).

29. Lopez, C.A., de Vries, A.H. & Marrink, S.J. Computational microscopy of cyclodextrin mediated cholesterol extraction from lipid model membranes. *Scientific reports* **3**, 2071 (2013).

30. Pediconi, M.F., Gallegos, C.E., De Los Santos, E.B. & Barrantes, F.J. Metabolic cholesterol depletion hinders cell-surface trafficking of the nicotinic acetylcholine receptor. *Neuroscience* **128**, 239-249 (2004).

31. Borroni, V. & Barrantes, F.J. Cholesterol modulates the rate and mechanism of acetylcholine receptor internalization. *J Biol Chem* **286**, 17122-17132 (2011).

32. Baier, C.J., Gallegos, C.E., Levi, V. & Barrantes, F.J. Cholesterol modulation of nicotinic acetylcholine receptor surface mobility. *European biophysics journal : EBJ* **39**, 213-227 (2010).

33. Barrantes, F.J. Cell-surface translational dynamics of nicotinic acetylcholine receptors. *Front Synaptic Neurosci* **6**, 25 (2014).

34. Kellner, R.R., Baier, C.J., Willig, K.I., Hell, S.W. & Barrantes, F.J. Nanoscale organization of nicotinic acetylcholine receptors revealed by stimulated emission depletion microscopy. *Neuroscience* **144**, 135-143 (2007).

35. Sadegh, S., Higgins, J.L., Mannion, P.C., Tamkun, M.M. & Krapf, D. Plasma Membrane is Compartmentalized by a Self-Similar Cortical Actin Meshwork. *Physical Review X* **7**, 011031 (2017).

36. Sikora, G., Burnecki, K. & Wylomanska, A. Mean-squared-displacement statistical test for fractional Brownian motion. *Phys Rev E* **95**, 032110 (2017).
